# Novel Potent and Selective Dopamine D4 Receptor Piperidine Antagonists as Potential Alternatives for the Treatment of Glioblastoma

**DOI:** 10.3390/ph18050739

**Published:** 2025-05-17

**Authors:** Federica Matteucci, Pegi Pavletić, Alessandro Bonifazi, Rian Garland, Hideaki Yano, Consuelo Amantini, Laura Zeppa, Emanuela Sabato, Giulio Vistoli, Valerio Mammoli, Loredana Cappellacci, Fabio Del Bello, Gianfabio Giorgioni, Riccardo Petrelli, Alessia Piergentili, Wilma Quaglia, Alessandro Piergentili

**Affiliations:** 1 Medicinal Chemistry Unit, School of Pharmacy, Chemistry Interdisciplinary Project (ChIP), University of Camerino, 62032 Camerino, Italy; federica.matteucci@unicam.it (F.M.); pegi.pavletic@biotech.uniri.hr (P.P.); loredana.cappellacci@unicam.it (L.C.); gianfabio.giorgioni@unicam.it (G.G.); riccardo.petrelli@unicam.it (R.P.); alessia.piergentili@unicam.it (A.P.); alessandro.piergentili@unicam.it (A.P.); 2 Faculty of Biotechnology and Drug Development, University of Rijeka, 51000 Rijeka, Croatia; 3 Department of Pharmacology and Toxicology, Center for Addiction Sciences and Therapeutics, University of Texas Medical Branch, Galveston, TX 77555, USA; albonifa@utmb.edu; 4 Department of Pharmaceutical Sciences, School of Pharmacy and Pharmaceutical Sciences, Bouvé College of Health Sciences, Center for Drug Discovery, Northeastern University, Boston, MA 02115, USA; garland.r@northeastern.edu (R.G.); h.yano@northeastern.edu (H.Y.); 5 Immunopathology and Molecular Medicine Unit, School of Biosciences and Veterinary Medicine, University of Camerino, 62032 Camerino, Italy; consuelo.amantini@unicam.it (C.A.); laura.zeppa@unicam.it (L.Z.); 6 Department of Pharmaceutical Sciences, University of Milan, 20133 Milano, Italy; emanuela.sabato@unimi.it (E.S.); giulio.vistoli@unimi.it (G.V.); 7 Center for Drug Discovery and Development-IDD, Aptuit, an Evotec Company, 37135 Verona, Italy; valerio.mammoli@evotec.com

**Keywords:** dopamine D4 receptors, potent and selective D4R antagonists, 4-butyl- and 4-benzyl-piperidines, docking studies, glioblastoma

## Abstract

**Background/Objectives**: D4R antagonists have recently been suggested as potential therapeutic alternatives to the standard treatments of glioblastoma (GBM). In this study, new piperidine-based ligands, analogs of the potent and selective D4R compounds 77-LH-28-1 (**7**) and its 4-benzyl analog **8**, were synthesized and studied to investigate the effects produced by variations in the distances between the pharmacophoric features on the D4R affinity and selectivity. **Methods**: All the new compounds **9–20** were evaluated for their radioligand binding affinity at D2-like receptor subtypes and the results were rationalized by docking studies and molecular dynamics (MD) simulations. The functional profiles of the most interesting derivatives were assessed at D4R Go and Gi protein and β-arrestin by BRET assay and their potential anticancer activity was determined in GBM cell lines. **Results**: Radioligand binding results highlighted that the derivatives bearing a terminal butyl chain showed structure–activity relationships different from those with a benzyl terminal. From functional studies performed on the best derivatives **12** and **16**, the response profiles of both compounds were more robust in antagonist mode, with derivative **16** showing higher antagonist potency than **12** across all three transducers. Interestingly, **12** and **16** dose-dependently decreased the cell viability of GBM cells, inducing cell death and cell cycle arrest, promoting an increase in ROS production, causing mitochondrial dysfunction, and significantly inhibiting colony formation. **Conclusions**: The promising biological profiles of **12** and **16** make them new lead candidates that warrant further investigation to gain a better understanding of the mechanism behind their antitumor activity and better evaluate their potential for GBM treatment.

## 1. Introduction

Dopamine receptors (DRs) are membrane proteins belonging to class-A metabotropic G-protein-coupled receptors, predominantly located in the central nervous system (CNS) of vertebrates. They play a role in various neurological functions such as pleasure, motivation, memory, learning, cognition, and coordination, as well as in neuroendocrine signaling modulation. Five dopamine receptor subtypes (D1R-D5R) with different structures, pharmacological effects, and locations in the brain have been identified so far and are grouped into two subfamilies, namely “D1-like” (comprising D1R and D5R) and “D2-like” (including D2R, D3R, and D4R) [1,2,3]. Studies using recombinant systems have shown that D2-like receptors have significantly higher affinity for dopamine compared to D1-like receptors, with a 10- to 100-fold difference. While D1-like receptors couple to Gα_s/olf_-proteins which stimulate adenylyl cyclase to produce cAMP, D2-like receptor activation decreases cAMP levels by coupling to inhibitory Gα_i/o_-proteins [2,4]. Furthermore, several other pathways, involving β-arrestins, ion channels, phospholipases, or protein kinases, were demonstrated to be affected by DRs through G-protein-dependent or -independent mechanisms [3,5].

Within the D2-like receptor family, D4R has recently gained attention as a promising target for treating various prevalent CNS conditions, such as alcohol and substance use disorders, L-DOPA-induced dyskinesia, attention deficit hyperactivity disorder, eating disorders, and brain cancers [6,7,8,9]. This subtype is known for its high genetic variability in the human genome, especially in the gene region that codes for the third intracellular loop (ICL3) of the receptor. The ICL3 of D4R harbors a 16-amino acid polypeptide with 2- to 11-repeat forms, where the most prevalent forms are 4-repeat (64%), followed by 7- and 2-repeat (21% and 8%, respectively). This variation can affect the interaction of D4R with adenylyl cyclase [6,10].

In the CNS, D4R is predominantly localized in the frontal cortex, hippocampus, amygdala, hypothalamus, medulla, and pituitary gland, indicating that it might play a crucial role in emotional, cognitive, and executive functions [6,11]. Moreover, an interesting relationship between D4R and glioblastoma (GBM) has been hypothesized and, specifically, D4R antagonists have recently been suggested as potential therapeutic alternatives to its standard treatments [12,13].

Most of the D4R ligands reported in the literature belong to different classical chemotypes, including dibenzodiazepines, piperazines, piperidines, and morpholines, which have extensively been described in previous reviews [6,9,14]. Additionally, further chemotypes have been identified by exploiting the resolved crystal structures of the antipsychotic drug nemonapride or the potent antagonist L-745,870 complexed with D4R (PDB ID = 5WIU or PDB ID = 6IQL, respectively) [15,16,17].

**Figure 1:**
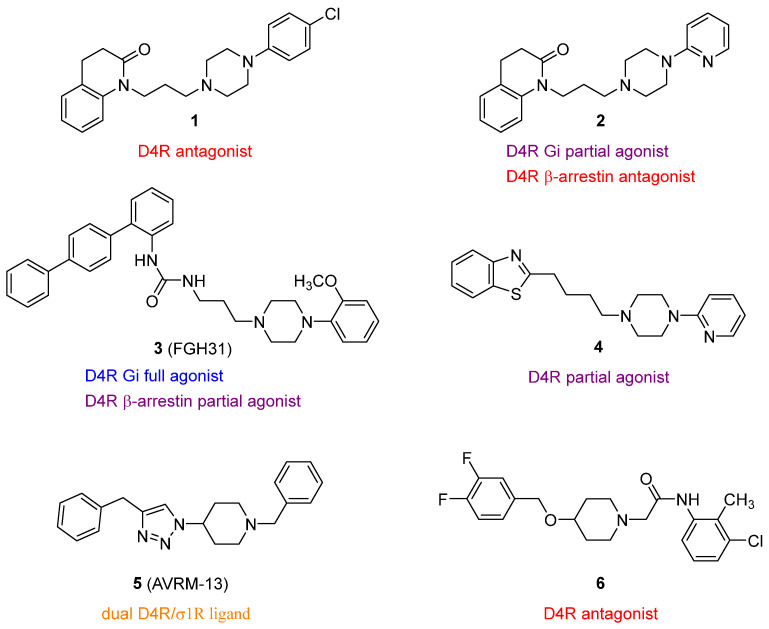
Chemical structures of the recently discovered D4R piperazines and piperidines **1–6**.

Among all chemotypes, piperazines and piperidines represent the most studied classes of D4R ligands, which share a common pharmacophore consisting of a lipophilic portion connected by a spacer to a basic function and an aromatic terminal. Such scaffolds also characterize the most recently reported D4R compounds. Specifically, concerning piperazines, in 2022, the potent and selective D4R antagonist **1** and the biased ligand **2** (Figure 1) were reported to decrease the viability of GBM cell lines [13]. In 2023, FGH31 (**3**, Figure 1) was identified as a D4R full agonist endowed with exceptional functional selectivity for Gi protein with respect to β-arrestin (bias factor = 306) [18] and **4** (Figure 1) as a potent D4R partial agonist, able to decrease cocaine self-administration in rats [8].

Regarding piperidines, subtype-selective ligands showing µM affinity for D4R were described in 2022. In particular, a dual D4R/σ1R profile has been reported for the 4-benzyltriazolylpiperidine AVRM-13 (**5**, Figure 1) [19], while the D4R selective 4-benzyloxypiperidine **6** (Figure 1) showed low clearance and good CNS penetration in rats [20].

**Figure 2:**
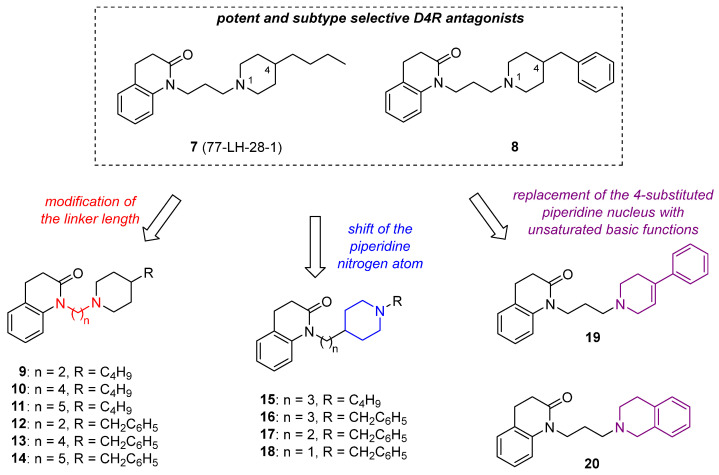
Chemical structures of the D4R ligands **7–20**.

We have also disclosed a promising series of D4R piperidines and piperazines inspired by the structure of the known muscarinic M1 bitopic agonist 77-LH-28-1 (**7**, Figure 2), which we demonstrated to behave as a potent and subtype-selective D4R ligand too. Such a compound is the first example of D4R-selective piperidines with a unique structural feature, consisting of an n-butyl substituent instead of an aromatic terminal [21]. Within the piperidines, the 4-benzyl derivative **8** (Figure 2) emerged as the most interesting compound, showing a D4R affinity and selectivity profile similarly to **7** (Table 1). In functional studies, they both behaved as antagonists in Gi activation and β-arrestin 2 recruitment assays [21].

The interesting results obtained with **7** and **8** prompted us to synthesize and study a series of structural analogs, with the aim of investigating the effects produced by variations in the distances between the pharmacophoric features, such as the quinolinone portion, the basic function, and the butyl substituent or the aromatic terminal, on D4R affinity and selectivity. Specifically, the following changes were designed: (i) modification of the linker length between the quinolinone nucleus and the basic function of **7** and **8** (compounds **9–11** and **12–14**, respectively, Figure 2); (ii) shift of the piperidine nitrogen atom from position 1 to position 4 of **7** (compound **15**) and **8** (compound **16**, whose interesting profile inspired the design of its lower homologues **17** and **18**, Figure 2); (iii) replacement of the 4-substituted piperidine nucleus with unsaturated basic functions (the 4-phenyl-1,2,3,6-tetrahydropyridine **19**, whose basic function is also present in the potent and selective D4R ligand RO-10-58-24 [6] and the 1,2,3,4-tetrahydroisoquinoline **20**, in which the piperidine ring is benzo-fused, Figure 2).

All the new compounds were evaluated for their D2R, D3R, and D4R affinity by radioligand binding assay and the functional profiles of the most interesting derivatives were also assessed at D4R Go and Gi proteins and β-arrestin by using the Bioluminescence Resonance Energy Transfer (BRET) assay. Docking studies helped to clarify the binding mode of this series of compounds. Lastly, considering the proposed involvement of D4R in GBM, the potential anticancer activity of the most interesting ligands was determined in U87 MG, T98G, and U251 MG glioma cells.

## 2. Results and Discussion

### 2.1. Synthesis of Compounds **9**–**20**

**Scheme 1:**
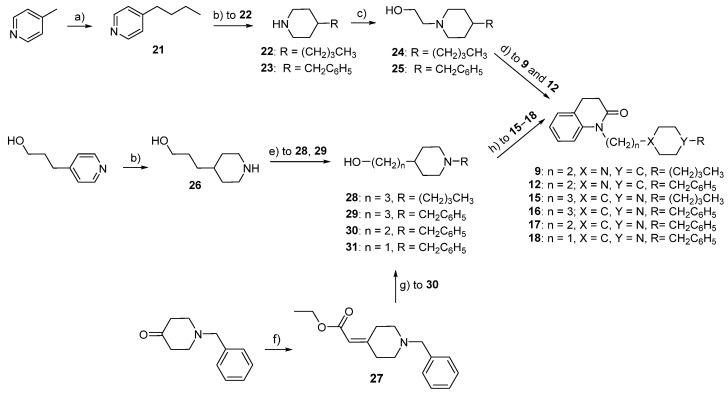
Synthesis of **9**, **12,** and **15–18**. Reagents and conditions: (a) CH_3_CH_2_CH_2_Br, *n*-BuLi, THF, 20 h, −78 °C/rt; (b) H_2_ 6 atm, Pt_2_O, 4 N HCl, CH_3_OH, 24 h, rt; (c) BrCH_2_CH_2_OH, K_2_CO_3_, CH_3_CN, 2 h, 95 °C; (d) . SOCl_2_, CHCl_3_, 3 h, reflux, then 3,4-dihydroquinolin-2(1*H*)-one, xylene, 18 h, 120 °C; (e) CH_3_(CH_2_)_3_Br for **28**, C_6_H_5_CH_2_Br for **29**, K_2_CO_3_, CH_3_CN, 3 h, reflux; (f) (C_2_H_5_O)_2_P(O)CH_2_CO_2_C_2_H_5_, K_2_CO_3_, THF, 24 h, reflux; (g) LiAlH_4_, THF, 20 h, reflux; (h) 48% HBr, 3 h, reflux, then. 3,4-dihydroquinolin-2(1*H*)-one, NaH, DMF, 18 h, rt.

Compounds **9**, **12**, and **15**–**18** were prepared according to the procedure reported in Scheme 1. Intermediate 4-butylpiperidine **22** [22], obtained by the reaction of 4-picoline with propyl bromide in the presence of *n*-butyllithium, followed by the hydrogenation of pyridine **21** at 6 atm in the presence of Adams’ catalyst, and 4-benzylpiperidine **23**, purchased from Merck Life Science S.r.l., Milan, Italy (142360), were treated with 2-bromoethanol in presence of potassium carbonate to afford alcohols **24** and **25**, respectively. Treatment with thionyl chloride converted **24** and **25** to the corresponding alkyl chlorides, which were reacted with 3,4-dihydroquinolin-2(1*H*)-one to give the desired compounds **9** and **12**, respectively.

**Scheme 2:**
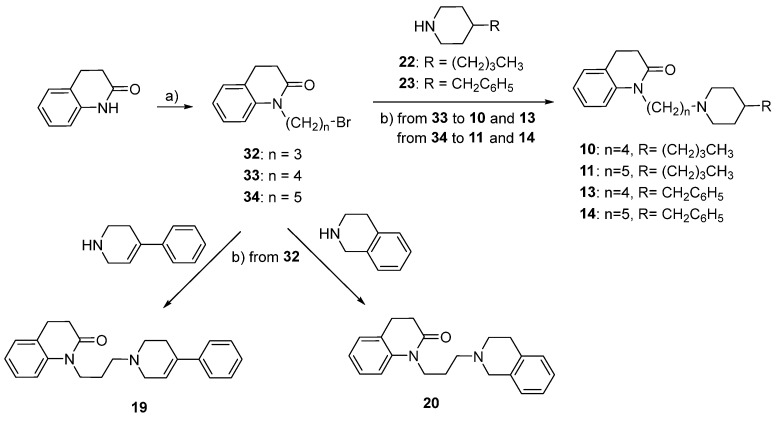
Synthesis of **10**, **11**, **13**, **14, 19,** and **20**. Reagents and conditions: (a) BrCH_2_CH_2_CH_2_Br for **32**, BrCH_2_CH_2_CH_2_CH_2_Br for **33**, BrCH_2_CH_2_CH_2_CH_2_CH_2_Br for **34**, NaH, DMF, 20 min, rt; (b) K_2_CO_3_, DMF, 4 h, 70 °C.

Alcohols **28** and **29** [23] were obtained by reduction of the commercially available 3-(pyridin-4-yl)propan-1-ol in the presence of Adams’ catalyst, followed by coupling of the piperidine **26** [24] with butyl bromide or benzyl bromide, respectively. Alcohol **30** was prepared by the reaction of 1-benzylpiperidin-4-one with triethyl phosphonoacetate, followed by the reduction of the α,β-unsaturated ester **27** with lithium aluminum hydride [25]. Alcohol **31** was purchased from Merck Life Science S.r.l., Milan, Italy (CDS011320). Treatment with 48% hydrobromic acid converted **28–31** to the corresponding alkyl bromides, which were reacted with 3,4-dihydroquinolin-2(1*H*)-one to give the final derivatives **15–18**, respectively. Compounds **10**, **11**, **13**, **14**, **19,** and **20** were prepared according to the procedure reported in Scheme 2. Treatment of 3,4-dihydroquinolin-2(1*H*)-one with either 1,3-dibromopropane or 1,4-dibromobutane or 1,5-dibromopentane in the presence of NaH 60% dispersion in mineral oil yielded the intermediates **32**, **33,** and **34**, respectively [13]. Coupling of bromides **33** or **34** with the proper piperidine **22** or **23** afforded the final compounds **10–14**, while the reaction of bromide **32** with the commercially available 4-phenyl-1,2,3,6-tetrahydropyridine or 1,2,3,4-tetrahydroisoquinoline yielded the desired products **19** and **20**, respectively.

### 2.2. Binding Studies

The radioligand binding affinity of the novel derivatives **9–20** was assessed with the human recombinant D2-like receptor subtypes (D2R, D3R, and D4R) stably expressed in HEK293 cell membranes by using the radioligand [^3^H]*N*-methylspiperone, according to previous procedures [26,27]. The p*K*_i_ values are reported in Table 1, along with those of leads **7** and **8**, included for useful comparison.

The results highlight that all the newly synthesized compounds show affinities for D4R higher than those for D2R and D3R, but with a high variability in p*K*_i_ values (from 6.12 to 9.18) as well as in D2/D4 and D3/D4 selectivity (from 5.8 to 2239 and from 1.4 to 1413, respectively).

Concerning the modification of the linker length between the quinolinone nucleus and the basic function of **7** and **8**, the elongation of the chain from 3 to 4 (**10** and **13**, respectively) and especially to 5 carbon atoms (**11** and **14**, respectively) is detrimental only for D4R affinity, with a consequent marked decrease in D2/D4 and D3/D4 selectivity. Instead, the shortening of the linker from a 3 to 2 carbon chain produces different effects. Indeed, the lower homolog of the lead **7** (compound **9**) shows significantly lower p*K*_i_ values for all the D2-like receptor subtypes. Interestingly, the same modification in **8**, affording **12**, slightly increases the affinity for D4R and maintains the good selectivity profile of the lead.

The shift of the piperidine nitrogen atom from position 1 to position 4 of **7** and **8**, yielding the regioisomers **15** and **16**, respectively, also differently affects D4R affinity and selectivity. Indeed, concerning **15**, a marked decrease in affinity especially for D4R (p*K*_i_ = 6.12) is observed, with a consequent drop in D2/D4 and D3/D4 selectivity (13 and 1.4, respectively), while **16** maintains the high D4R affinity (p*K*_i_ = 8.79) of the lead **8** and even shows higher D2/D4 selectivity (2239 vs. 724) due to a reduced affinity for D2R. The result obtained with **15** confirms the above observation that the increase in the distance between the quinolinone nucleus and the basic function is detrimental to D4R interaction, whereas the high D4R affinity of **16** is surprising and prompted us to prepare and study its lower homologues **17** and **18**. They both show D4R p*K*_i_ values of about two orders of magnitude lower than **16**, suggesting that the shortening of its chain from 3 to 2 and 1 carbon atoms is detrimental to D4R affinity.

The overall results highlight that the derivatives bearing a terminal butyl chain show structure–activity relationships (SARs) different from those with a benzyl terminal, suggesting that the two series of compounds interact with D4R through different binding modes.

Finally, concerning the replacement of the 4-substituted piperidine nucleus with unsaturated basic functions, the 4-phenyl-1,2,3,6-tetrahydropyridine **19** displays a good D4R affinity (p*K*_i_ = 8.82) and selectivity profile (D2/D4 = 380, D3/D4 = 162), comparable to those of its saturated analog (D4R p*K*_i_ = 8.71; D2/D4 = 234 D3/D4 = 363) as we previously reported [21]. Instead, a drop in D4R affinity and selectivity is observed with the 1,2,3,4-tetrahydroisoquinoline **20**, suggesting that the fusion between the piperidine nucleus and the phenyl ring is detrimental.

### 2.3. Functional Studies

The Bioluminescence Resonance Energy Transfer (BRET)-based assay was used to assess the D4R functional activity of compounds **12** and **16**, selected for their noteworthy affinity/selectivity profiles. Specifically, both D4R Go and Gi activation as well as D4R β-arrestin 2 recruitment were evaluated to better characterize the functional profile of the ligands. Potency and efficacy, expressed as pEC_50_ and Emax, respectively, are reported in Table 2, together with those of the neurotransmitter dopamine, included as a reference compound.

Pretreatment with a fixed amount of dopamine (1 μM) allowed us to also assay the antagonist effects of the compounds, whose pIC_50_ and Imax values are reported in Table 2, along with those of the reference antagonist L745,870.

From the data analysis, it emerges that the response profiles of the test compounds are generally more robust in antagonist mode, though **16** also behaves as a weak partial agonist (E_max_ > 20%) at D4R Go, with potency comparable to that of dopamine (pEC_50_ = 8.36). When tested in antagonist mode, compound **12** has similar potency across transducers, with a slightly lower inhibition efficacy at β-arrestin 2. Derivative **16** shows a significantly higher inhibition potency than **12** across all three transducers, with its inhibition efficacy slightly higher at Gi than Go protein and β-arrestin 2. Notably, **16** has higher inhibition potency than L745,870 at β-arrestin 2 despite its inhibition efficacy lower than L745,870.

### 2.4. Molecular Modeling Studies

**Figure 3:**
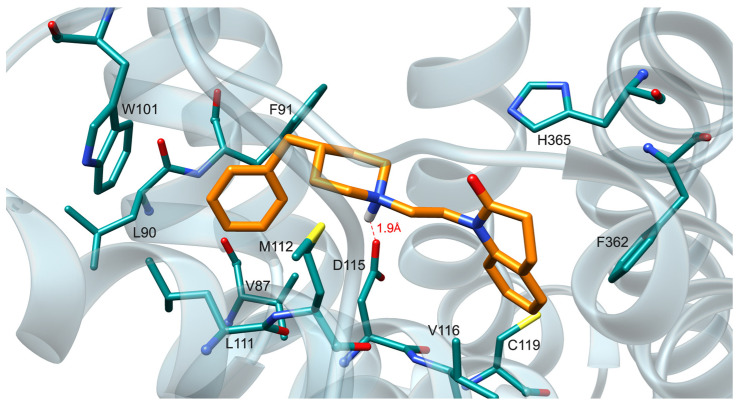
Key interactions stabilizing the putative complex of **12** (in orange) with D4R (ChemPLP score = −105.72 kcal/mol). The protein is colored in light blue and its key interacting residues are highlighted in sticks (oxygen atoms in red, nitrogen atoms in blue and sulfur atoms in yellow); hydrogen bond is depicted in a red dotted line with its distance expressed in Ångström.

To rationalize the obtained results on D4R, molecular docking studies were carried out using the resolved D4R structure in complex with nemonapride (PDB id: 5WIU). Figure 3 shows the key interactions stabilizing the putative complex of compound **12** with D4R and emphasizes the key role played by π-π stackings, which involve both the quinolinone ring with Trp358, Phe361, and Phe362 and the phenyl ring with Phe91 and Trp101. Along with the pivotal ion pair with Asp115, reinforced by the H-bond with Tyr389, the carbon skeleton of the piperidine ring and the alkyl linker are engaged in hydrophobic contacts with Met112 and Leu187. Taken globally, Figure 3 confirms that shortening the linker between piperidine and quinolinone does not affect the molecular binding since the richness of the aromatic side chains surrounding the ligands allows the quinolinone and phenyl moieties to roughly maintain their key π-π stackings without hampering the key ion pair with Asp115.

**Figure 4:**
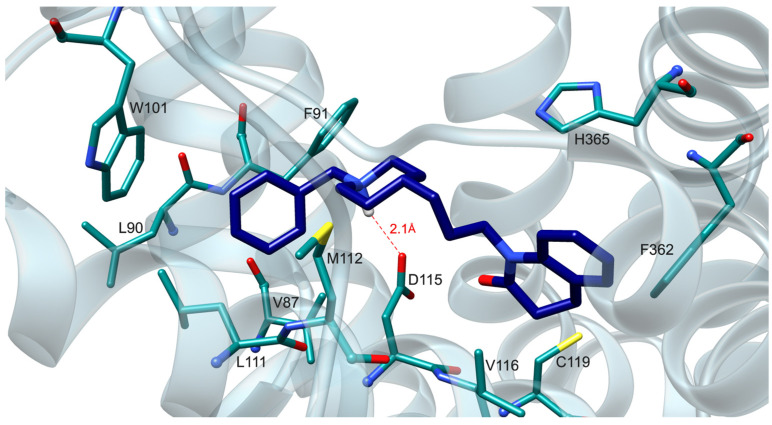
Key interactions stabilizing the putative complex of **16** (in blue) with D4R (ChemPLP score = −107.01 kcal/mol). The protein is colored in light blue and its key interacting residues are highlighted in sticks (oxygen atoms in red, nitrogen atoms in blue and sulfur atoms in yellow); hydrogen bond is depicted in a red dotted line with its distance expressed in Ångström.

The rather precise size of the binding pocket accommodating the two aromatic moieties can, in turn, explain why extending the linker plays a negative role by moving the protonated amine away from Asp115. Figure 4 also reveals that the mentioned adaptability of the π-π stackings can rationalize why the piperidine nitrogen atom in position 4 is well tolerated, since ligand **16** retains all the hydrophobic/aromatic interactions with the minimal shift in the protonated amine, which does not affect its capacity to contact Asp115.

**Figure 5:**
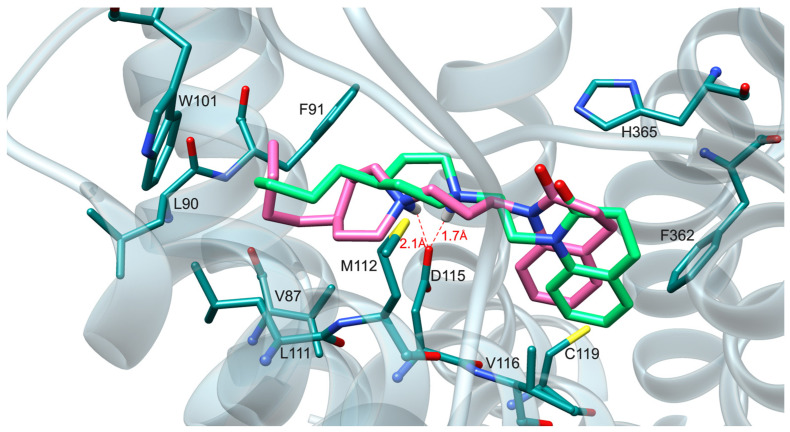
Comparison of the binding mode for lead **7** (in pink, ChemPLP score = −100.30 kcal/mol) and **9** (in green, ChemPLP score = −95.65 kcal/mol). Note how the linker shortening affects the accommodation of both quinolone and piperidine ring. The protein is colored in light blue and its key interacting residues are highlighted in sticks (oxygen atoms in red, nitrogen atoms in blue and sulfur atoms in yellow); hydrogen bond is depicted in a red dotted line with its distance expressed in Ångström.

On the contrary, Figure 5 compares the putative binding modes for the lead compound **7** and its lower homolog **9** and highlights that the shorter linker prevents an optimal accommodation of all the interacting moieties. The different effect of the linker shortening between 4-benzyl and 4-butyl derivatives can be explained by considering that while the aromatic side chains homogeneously surround the binding pocket (as mentioned above), the alkyl side chains are focused on a rather narrow region of the pocket (e.g., Val87, Leu90, Leu111, and Met112). This reduces the ligand adaptability, and compound **9** can roughly maintain the contacts elicited by the protonated amine and quinolone ring while losing the most apolar contacts stabilized by the piperidine ring and alkyl chain.

**Figure 6:**
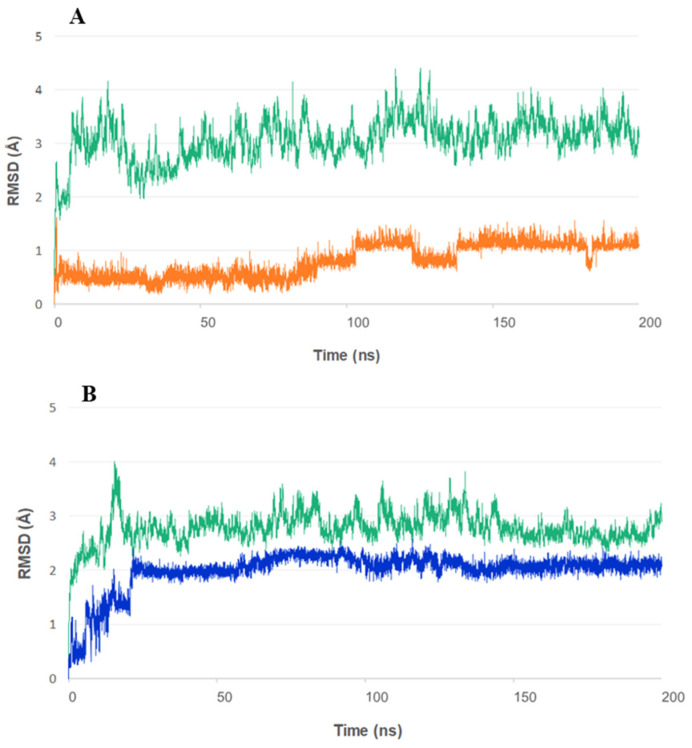
RMSD profiles based on the MD simulations of D4R in complex with compound **12** ((**A**), backbone in green and ligand in orange) and with compound **16** ((**B**), backbone in green and ligand in blue).

Next, molecular dynamics (MD) simulations were performed to optimize and assess the putative complexes for compounds **12** and **16**. The resulting root mean square deviation (RMSD) profiles for the protein backbone and the sole ligand of the two complexes were computed and plotted in Figure 6.

In detail, the protein exhibited reasonably stable behaviors in both simulations, after an initial short equilibration (20–25 ns), during which the protein backbone experiences some slight fluctuations. In contrast, the two ligands showed some differences in their RMSD profiles. Compound **12** retained low and constant RMSD values during the entire MD run, while the RMSD of compound **16** revealed a sharp increase in the first 20 ns to remain stable during the rest of the simulation. A visual analysis of these initial frames highlighted a shift in the arrangement of the ligand’s quinolinone moiety to allow for the interaction between its carbonyl group and Arg186.

The root mean square fluctuation (RMSF) of the backbone atoms was also analyzed for both simulations, showing analogous behaviors. A certain degree of flexibility has been highlighted around residues 145–148, 172–182, 246–250, 277–282, 306–313, which correspond to loop regions, while all transmembrane domains and the binding site region maintained stability well. Taken together, the behavior of the complexes during the simulations highlighted the overall stability of the above-mentioned interactions, thus confirming the reliability of the computed poses.

### 2.5. Biological Studies in GBM Cell Lines

To evaluate the potential antitumor effects of the D4R antagonists **12** and **16** on GBM, we first performed the sulforhodamine B (SRB) assay on U87 MG, T98G, and U251 MG glioma cells treated for 48 h with the test compounds. The data demonstrate that both compounds promote a significant reduction in cell viability in a dose-dependent manner in all three glioma cell lines. The ability of **12** and **16** to inhibit cell growth after 48 h of treatment is higher than that induced by temozolomide (TMZ), the only drug currently approved for the first-line treatment of GBM (Table 3 and Figure S1). For subsequent experiments, the compounds were tested at doses corresponding to their GI_50_ values.

**Figure 7:**
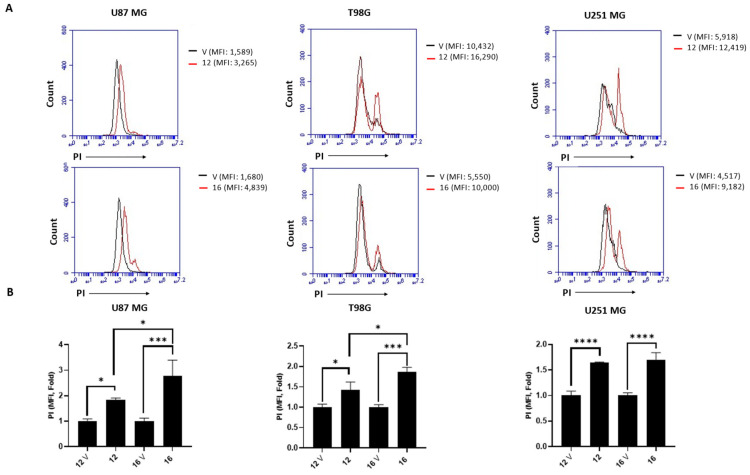
Cell death in glioma cells induced by compounds **12** and **16**. (**A**) Cell death was assessed in glioma cells treated for 48 h with compounds **12**, **16,** or vehicle by PI staining and evaluating the MFI of red fluorescence. Images are representative of three separate experiments. (**B**) Glioma cells were treated for 24 h and 48 h with compounds **12**, **16,** or vehicle (V). PI fluorescence (MFI) was expressed in folds with respect to the vehicle-treated cells. Data represent the mean ± SEM of three separate experiments. * *p* < 0.05; *** *p* < 0.001; ****p < 0.0001.

The decrease in the cell viability of tumor cells may be the consequence of two physiological processes, specifically the induction of cell death or the blockage of cell cycle progression [28]. To better elucidate the mechanisms of drug action, cytofluorimetric analysis and propidium iodide (PI) staining were employed to assess cell death in glioma cells treated with compounds **12** and **16**. Figure 7 shows a marked increase in PI red fluorescence, as highlighted by the mean fluorescence intensity (MFI) values, evident in all glioma cells after treatment with both compounds, indicating the induction of cell death.

Moreover, to obtain an initial idea of the ability of these compounds to induce apoptosis, we also evaluated early phosphatidylserine exposure on the outer surface of the plasma membrane in glioma cells treated for 24 h with compounds **12** or **16**. The bi-parametric cytofluorimetric analysis, measured by Annexin V and PI fluorescence, shows that with respect to the vehicle, both compounds slightly increase the percentage of single Annexin V positive cells in U87 MG and T98G glioma lines, suggesting the induction of apoptosis, whereas an enhancement of single PI positive cells in U251 MG, indicating necrosis, is shown (Figure S2). Of course, additional experiments are necessary to better investigate and understand in detail the type of cell death induced by compounds **12** and **16**.

Gliomas represent a varied group of brain tumors characterized by high heterogeneity associated with resistance to current therapies, and it is well known that glioma cell plasticity leads to the acquisition of resistance to apoptosis [29]. For this reason, we used three glioma cell lines with different genetic backgrounds to better represent the tumor diversity and the response to therapy. The induction of apoptosis is no longer considered the only useful approach in cancer treatment. In fact, it has recently been accepted that inducing programmed necrosis, which is caspase-independent and associated with ROS overproduction and mitochondria impairment, could also improve therapeutic strategies to kill glioma cells [30,31,32]. Our preliminary results are in line with this recent evidence, and compounds **12** and **16** could be useful to promote different cell death mechanisms, overcoming the problem of glioma phenotypic heterogeneity.

**Figure 8:**
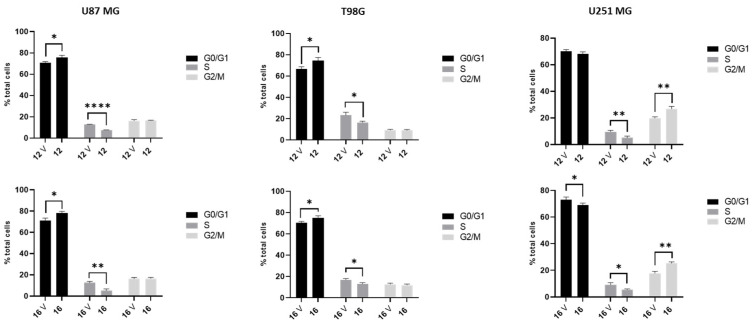
Compounds **12** and **16** induce cell cycle impairment. The cell cycle was analyzed by cytofluorimetric analysis and the percentage of cells in each cell cycle phase was measured. Data represent the mean ± SEM of three separate experiments. * *p* < 0.05; ** *p* < 0.01; **** *p* < 0.0001.

Our results also demonstrate that the treatment impairs the cell cycle and, specifically, compound **12** modifies the percentage of cells in the S phase, whereas compound **16** changes the G0/G1 and the S phases in all three glioma cell lines with respect to the vehicle-treated cells (Figure 8). In addition, both compounds promote an increase in the percentage of U251 MG cells in the G2/M phase. Different responses to treatment are expected in glioma because the lines differ in phenotypic characteristics such as proliferation, invasion, and migration, and these three cell lines in particular have different p53 and MGMT statuses.

**Figure 9:**
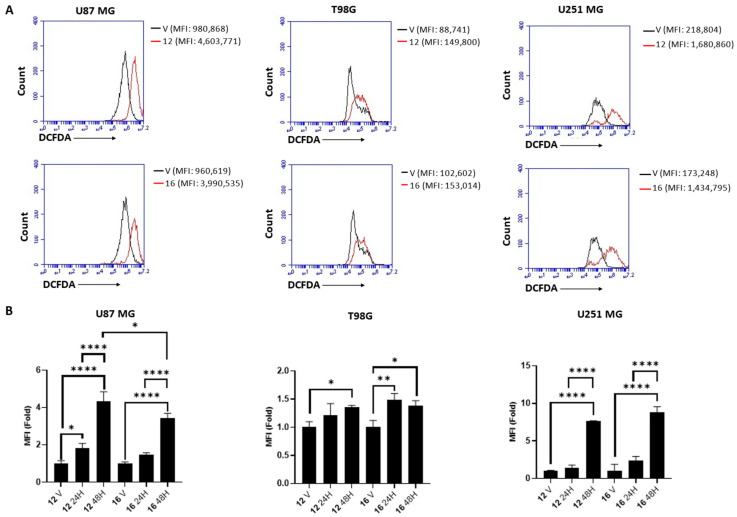
ROS production induced by compounds **12** and **16**. (**A**) ROS production was assessed by 2’,7’-dichlorodihydrofluorescein diacetate (DCFDA) staining and cytofluorimetric analysis in glioma cells treated with compounds **12** and **16** or with vehicle (V) for 24 h. Green fluorescence expressed as MFI was evaluated. Images are representative of three separate experiments. (**B**) MFI of the DCFDA fluorescence was expressed in folds with respect to vehicle-treated cells. Data represent the mean ± SEM of three separate experiments. * *p* < 0.05; ** *p* < 0.01; **** *p* < 0.0001.

Given that cell death and cell cycle blockage are usually associated with the enhancement of reactive oxygen species (ROS) production [33], we investigated the increase in ROS generation in glioma cells treated with compounds **12** and **16** for 24 h and 48 h. The results show that both compounds promote an increase in ROS production in glioma cells (Figure 9A), and specifically in a time-dependent manner in U87 MG, after 48 h of treatment in U251 MG and at similar levels at both times of treatment in T98G cells (Figure 9B).

**Figure 10:**
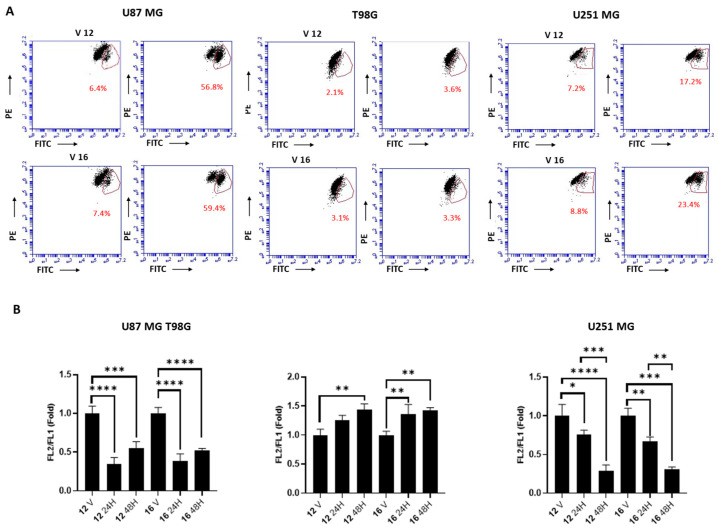
Mitochondrial dysfunction in glioma cells promoted by treatment with compounds **12** and **16**. (**A**) Mitochondrial dysfunction was investigated by JC-1 staining and cytofluorimetric analysis. Mitochondrial potential membrane was assessed in glioma cells treated with compounds **12** and **16** or with vehicle (V) for 24 h. Red gate represents cell subpopulation with mitochondrial depolarization. Red numbers indicate the cell percentage. Images are representative of three separate experiments. (**B**) FL2/FL1 ratio was calculated to evaluate changes in mitochondrial membrane potential in glioma cells treated with compounds **12**, **16,** or vehicle for 24 and 48 h. FL2/FL1 ratio was expressed as folds with respect to vehicle-treated cells. Data represent the mean ± SEM of three separate experiments. * *p* < 0.05; ** *p* < 0.01; *** *p* < 0.001; **** *p* < 0.0001.

ROS enhancement is associated with mitochondrial dysfunction, and mitochondrial-targeted therapies represent promising strategies for treating cancers including GBM [34]. Thus, we also evaluated the mitochondrial impairment in glioma cells treated with **12** and **16** for 24 and 48 h by using JC-1 staining. A significant drop in the mitochondrial potential membrane, found to be associated with glioma cell death [35], was demonstrated in U87 MG and U251 MG cells after treatment with both compounds (Figure 10A), as shown by the increase in the percentage of cells with improved green fluorescence (FL-1, Figure 10A) and by the consequent marked reduction in the FL2/FL1 ratio (Figure 10B). Instead, compounds **12** and **16** provoke an increase in the FL2/FL1 ratio with respect to the vehicle in T98G cells, indicating an evident mitochondrial hyperpolarization (Figure 10A,B). It is well known that the presence of hyperpolarized mitochondria is common in cancers and is often associated with enhanced invasiveness [36]. However, the induction of mitochondrial hyperpolarization is responsible for cell cycle arrest [37,38], and hyperpolarization is also useful to enhance the selective transfer of different mitochondria-targeting drugs into cancer cells [39].

**Figure 11:**
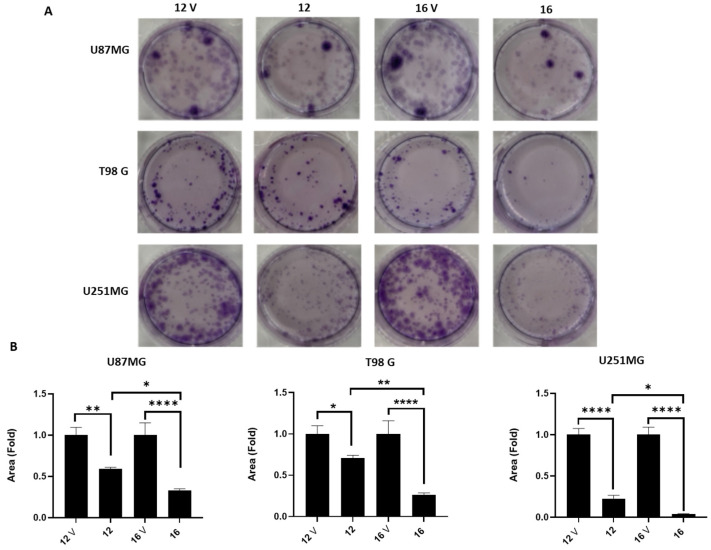
Inhibition of the colony formation induced by treatment with compounds **12** and **16**. (**A**) The colony formation assay showing colony inhibition in response to treatment for 48 h and after 14 days from seeding. Representative images of the macroscopic visualization of the crystal violet-stained colonies. (**B**) Results of the colony formation assay by analyzing the stained area. Data represent the mean ± SEM of three separate experiments. * *p* < 0.05; ** *p* < 0.01; **** *p* < 0.001.

To investigate cells that survive over time and retain proliferative capacity, we finally performed a colony formation assay in glioma cells treated with the vehicle or compounds **12** and **16** for 48 h and then cultured in fresh medium for an additional 14 days. The colony formation was significantly inhibited by both compounds and, in particular, compound **16** showed greater effects compared with **12** in all glioma cell lines (Figure 11A,B). Overall, these results demonstrate that, after treatment, only a very small fraction of seeded glioma cells retained the capacity to produce colonies.

## 3. Materials and Methods

### 3.1. Chemistry

The equipment for compound preparation and characterization is reported in the Supplementary Materials. The elemental analysis results for the final compounds **9**–**20** are reported in Table S1.

#### 3.1.1. 1-(2-(4-Butylpiperidin-1-yl)ethyl)-3,4-dihydroquinolin-2(1*H*)-one (**9**)

SOCl_2_ (0.3 g, 2.5 mmol) was added to a solution of **24** (0.2 g, 1.1 mmol) in CHCl_3_ (15 mL) and the mixture was stirred for 3 h at reflux. Then, it was cooled to 25 °C and the solvent was removed under reduced pressure to give a residue, which was crystallized from acetonitrile. The salt was diluted with xylene and the mixture was basified with NaOH at 0 °C. The organic layer was separated, dried over K_2_CO_3_, and filtered, and the solution was added dropwise to a suspension of NaH (0.16 g of 60% dispersion in mineral oil, 4 mmol) and 3,4-dihydroquinolin-2(1*H*)-one (0.15 g, 1 mmol) in xylene (10 mL) at 0 °C. After stirring for 18 h at 120 °C, the mixture was cooled at rt, diluted with H_2_O, and extracted with EtOAc (3 × 20 mL). The organic layer was washed with brine, dried over anhydrous Na_2_SO_4_, and concentrated under reduced pressure to afford a residue which was purified by column chromatography eluting with CHCl_3_/MeOH (99:1) to give an oil (33% yield). ^1^H NMR (DMSO, 293 K): δ 0.87 (t, *J* = 5.9 Hz, 3H); 1.25–1.56 (m, 9H); 1.82 (m, 2H); 2.61 (m, 2H); 2.88 (m, 2H); 2.98 (m, 2H); 3.21 (m, 2H); 3.52 (m, 2H); 4.26 (m, 2H); 6.96–7.31 (m, 4H). The free base was treated with oxalic acid to give the corresponding oxalate salt, which was crystallized from 2-PrOH. A white solid was obtained: mp 146–147 °C, ESI/MS *m*/*z*: 316 [M + H]^+^. Anal. Calcd (C_20_H_30_N_2_O.C_2_H_2_O_4_) C, H, N.

#### 3.1.2. 1-(4-(4-Butylpiperidin-1-yl)butyl)-3,4-dihydroquinolin-2(1*H*)-one (**10**)

A solution of **33** (0.42 g, 1.5 mmol) in DMF (10 mL) was added to a suspension of **22** (0.21 g, 1.7 mmol) and K_2_CO_3_ (0.30 g, 2.2 mmol) in DMF (10 mL). The reaction mixture was stirred for 4 h at 70 °C. Then, it was diluted with brine and extracted with EtOAc (3 × 50 mL). The organic layer was washed with brine and dried over anhydrous Na_2_SO_4_. The evaporation of the solvent under reduced pressure afforded a residue, which was purified by flash chromatography with gradient elution from cyclohexane: EtOAc (9:1) to 100% EtOAc to give a colorless oil (41% yield). ^1^H NMR (CDCl_3_, 293 K): δ 0.92 (t, *J* = 5.9 Hz, 3H); 1.36 (m, 9H); 1.83 (m, 6H); 1.98 (m, 2H); 2.47 (m, 2H); 2.68 (m, 2H); 2.90 (m, 2H); 2.99 (m, 2H); 3.97 (m, 2H); 6.96–7.31 (m, 4H). The free base was treated with oxalic acid to give the corresponding oxalate salt, which was crystallized from 2-PrOH. A white solid was obtained: mp 147–148 °C, ESI/MS m/z: 426 [M + H]^+^, 448 [M + Na]^+^. Anal. Calcd (C_22_H_34_N_2_O.C_2_H_2_O_4_) C, H, N.

#### 3.1.3. 1-(5-(4-Butylpiperidin-1-yl)pentyl)-3,4-dihydroquinolin-2(1*H*)-one (**11**)

The procedure described for **10** was used to prepare **11** starting from **22** and **34**. An oil was obtained (47% yield). ^1^H NMR (CDCl_3_, 293 K): δ 0.90 (t, *J* = 6.5 Hz, 3H); 1.22–1.43 (m, 9H); 1.83(m, 6H); 1.69 (m, 2H); 2.07 (m, 2H); 2.45 (m, 2H); 2.65 (m, 2H); 2.90 (m, 2H); 3.06 (m, 2H); 3.96 (m, 2H); 6.98–7.26 (m, 4H). The free base was treated with oxalic acid to give the corresponding oxalate salt, which was crystallized from 2-PrOH. A white solid was obtained: mp 133–134 °C, ESI/MS *m*/*z*: 357 [M + H]^+^, 379 [M + Na]^+^. Anal. Calcd (C_23_H_36_N_2_O.C_2_H_2_O_4_) C, H, N.

#### 3.1.4. 1-(2-(4-Benzylpiperidin-1-yl)ethyl)-3,4-dihydroquinolin-2(1*H*)-one (**12**)

The procedure described for **9** was used to prepare **12** starting from **25**. An oil was obtained (27% yield). ^1^H NMR (CDCl_3_, 293 K): δ 1.92 (m, 2H); 1.56–1.72 (m, 3H); 2.09 (m, 2H); 2.54–2.66 (m, 6H); 2.94 (m, 2H); 3.00 (m, 2H); 4.08 (m, 2H); 6.96–7.29 (m, 9H). A white solid was obtained: mp 152–153 °C, ESI/MS *m*/*z*: 349 [M + H]^+^, 371 [M + Na]^+^. Anal. Calcd (C_23_H_28_N_2_O.C_2_H_2_O_4_) C, H, N.

#### 3.1.5. 1-(4-(4-Benzylpiperidin-1-yl)butyl)-3,4-dihydroquinolin-2(1*H*)-one (**13**)

The procedure described for **10** was used to prepare **13** starting from **23** and **33**. An oil was obtained (53% yield). ^1^H NMR (CDCl_3_, 293 K): δ 1.42–1.79 (m, 9H); 2.09 (m, 2H); 2.46 (m, 2H); 2.50 (m, 2H); 2.62 (m, 2H); 2.89 (m, 2H); 3.05 (m, 2H); 3.98 (m, 2H); 6.93–7.41 (m, 9H). The free base was treated with oxalic acid to give the corresponding oxalate salt, which was crystallized from 2-PrOH. A white solid was obtained: mp 165–166 °C, ESI/MS *m*/*z*: 426 [M + H]^+^, 448 [M + Na]^+^. Anal. Calcd (C_25_H_32_N_2_O.C_2_H_2_O_4_) C, H, N.

#### 3.1.6. 1-(5-(4-Benzylpiperidin-1-yl)pentyl)-3,4-dihydroquinolin-2(1*H*)-one (**14**)

The procedure described for **10** was used to prepare **14** starting from **23** and **34**. An oil was obtained (68% yield). ^1^H NMR (CDCl_3_, 293 K): δ 1.39 (m, 2H); 1.41–1.78 (m, 9H); 2.09 (m, 2H); 2.46 (m, 2H); 2.49 (m, 2H); 2.62 (m, 2H); 2.83 (m, 2H); 3.10 (m, 2H); 3.91 (m, 2H); 6.93–7.38 (m, 9H). The free base was treated with oxalic acid to give the corresponding oxalate salt, which was crystallized from 2-PrOH. A white solid was obtained: mp 141–143 °C, ESI/MS *m*/*z*: 426 [M + H]^+^, 448 [M + Na]^+^. Anal. Calcd (C_26_H_34_N_2_O.C_2_H_2_O_4_) C, H, N.

#### 3.1.7. 1-(3-(1-Butylpiperidin-4-yl)propyl)-3,4-dihydroquinolin-2(1*H*)-one (**15**)

Alcohol **28** (0.2 g, 1 mmol) was dissolved in 48% aq HBr (1.1 mL, 20 mmol) and the mixture was stirred at reflux for 3 h. Then, it was cooled to rt, diluted with H_2_O (15 mL), and 28% aq NH_3_ (2 mL) was added at 0 °C. The mixture was extracted with *n*-hexane/Et_2_O (1:1 *v*/*v*, 2 × 50 mL). The organic layer was washed with brine (2 × 30 mL), dried over Na_2_SO_4_, and the solvent was removed under reduced pressure. The residue was diluted with DMF (5 mL) and the resulting solution was added dropwise to a suspension of 60% NaH dispersion in mineral oil (0.16 g, 4 mmol) and 3,4-dihydroquinolin-2(1*H*)-one (0.15 g, 1 mmol) in DMF (10 mL) at 0 °C. After stirring for 18 h at 120 °C, the mixture was cooled to rt, diluted with H_2_O, and extracted with EtOAc (3 × 20 mL). The organic layer was washed with brine (5 × 20 mL), dried over anhydrous Na_2_SO_4_, and concentrated under reduced pressure to afford a residue which was purified by column chromatography eluting with EtOAc/MeOH (98:2) to give an oil (28% yield). ^1^H NMR (CDCl_3_, 293 K): δ 0.95 (t, *J* = 5.9 Hz, 3H); 1.42 (m, 7H); 1.52 (m, 2H); 1.70 (m, 4H); 1.96 (m, 2H); 2.37 (m, 2H); 2.65 (m, 2H); 2.97 (m, 2H); 3.02 (m, 2H); 3.89 (m, 2H); 6.96–7.31 (m, 4H). The free base was treated with oxalic acid to give the corresponding oxalate salt, which was crystallized from 2-PrOH. A white solid was obtained: mp 91–92 °C, ESI/MS *m*/*z*: 316 [M + H]^+^. Anal. Calcd (C_21_H_32_N_2_O.C_2_H_2_O_4_) C, H, N.

#### 3.1.8. 1-(3-(1-Benzylpiperidin-4-yl)propyl)-3,4-dihydroquinolin-2(1*H*)-one (**16**)

The procedure described for **15** was used to prepare **16** starting from **29**. An oil was obtained (23% yield). ^1^H NMR (CDCl_3_, 293 K): δ 1.36 (m, 5H); 1.63 (m, 4H); 1.97 (m, 2H); 2.36 (m, 2H); 2.62 (m, 2H); 2.98 (m, 4H); 3.51 (s, 2H); 3.94 (m, 2H); 6.97–7.44 (m, 9H). The free base was treated with oxalic acid to give the corresponding oxalate salt, which was crystallized from 2-PrOH. A white solid was obtained: mp 183–184 °C, ESI/MS *m*/*z*: 363 [M + H]^+^, 386 [M + Na]^+^. Anal. Calcd (C_24_H_30_N_2_O.C_2_H_2_O_4_) C, H, N.

#### 3.1.9. 1-(2-(1-Benzylpiperidin-4-yl)ethyl)-3,4-dihydroquinolin-2(1*H*)-one (**17**)

The procedure described for **15** was used to prepare **17** starting from **30**. An oil was obtained (16% yield). ^1^H NMR (CDCl_3_, 293 K): δ 0.95–1.65 (m, 5H); 1.90 (m, 2H); 2.38 (m, 2H); 2.65 (m, 2H); 2.97 (m, 2H); 3.10 (m, 2H); 3.46 (m, 2H); 3.99 (m, 2H); 6.96–7.48 (m, 9H). The free base was treated with oxalic acid to give the corresponding oxalate salt, which was crystallized from 2-PrOH. A white solid was obtained: mp 175–176 °C, ESI/MS *m*/*z*: 349 [M + H]^+^, 371 [M + Na]^+^. Anal. Calcd (C_23_H_28_N_2_O.C_2_H_2_O_4_) C, H, N.

#### 3.1.10. 1-((1-Benzylpiperidin-4-yl)methyl)-3,4-dihydroquinolin-2(1*H*)-one (**18**)

The procedure described for **15** was used to prepare **18** starting from **31**. An oil was obtained (26% yield). ^1^H NMR (CDCl_3_, 293 K): δ 0.91 (m, 1H); 1,22 (m, 3H); 1.59 (m, 2H); 1.71 (m, 1H); 1.97 (m, 2H); 2.62 (m, 2H); 2.91 (m, 4H); 3.91 (m, 2H); 6.98–7.41 (m, 9H). The free base was treated with oxalic acid to give the corresponding oxalate salt, which was crystallized from 2-PrOH. A white solid was obtained: mp 103–104 °C, ESI/MS *m*/*z*: 335 [M + H]^+^, 357 [M + Na]^+^. Anal. Calcd (C_22_H_26_N_2_O.C_2_H_2_O_4_) C, H, N.

#### 3.1.11. 1-(3-(4-Phenyl-3,6-dihydropyridin-1(2*H*)-yl)propyl)-3,4-dihydroquinolin-2(1*H*)-one (**19**)

The procedure described for **10** was used to prepare **19** starting from **32** and 4-phenyl-1,2,3,6-tetrahydropyridine (39% yield). ^1^H NMR (DMSO, 293 K): δ 1.98 (m, 2H); 2.45- 2.56 (m, 6H); 2.90 (m, 2H); 3.05 (m, 2H); 3.70 (m, 2H); 3.99 (m, 2H); 6.19 (m, 1H); 7.02–7.49 (m, 9H). A white solid was obtained: mp 199–200 °C, ESI/MS *m*/*z*: 347 [M + H]^+^. Anal. Calcd (C_23_H_26_N_2_O.C_2_H_2_O_4_) C, H, N.

#### 3.1.12. 1-(3-(3,4-Dihydroisoquinolin-2(1*H*)-yl)propyl)-3,4-dihydroquinolin-2(1*H*)-one (**20**)

The procedure described for **10** was used to prepare **20** starting from **32** and 1,2,3,4-tetrahydroisoquinoline. An oil was obtained (46% yield). ^1^H NMR (CDCl_3_, 293 K): δ 1.99 (m, 2H); 2.60 (m, 6H); 2.72 (m, 2H); 2.98 (m, 2H); 3.69 (m, 2H); 4.03 (m, 2H); 6.89–7.32 (m, 8H). The free base was treated with oxalic acid to give the corresponding oxalate salt, which was crystallized from 2-PrOH. A white solid was obtained: mp 203–204 °C, ESI/MS *m*/*z*: 426 [M + H]^+^, 448 [M + Na]^+^. Anal. Calcd (C_21_H_24_N_2_O.C_2_H_2_O_4_) C, H, N.

#### 3.1.13. 4-Butylpyridine (**21**)

Butyllithium (16.8 mL, 42.00 mmol) was added dropwise to a solution of 4-picoline (3 g, 32 mmol) in dry THF (20 mL) at −78 °C. The reaction was allowed to warm up to rt and stirred for 2 h. The solution was then added dropwise to a solution of 1-bromopropane (5 mL, 64 mmol) in THF (10 mL) at −78 °C. The reaction was allowed to gradually warm up to rt and stirred for 20 h. H_2_O (1.5 mL) was added and the solvent was evaporated under reduced pressure to give a residue which was purified by flash chromatography eluting with cyclohexane/EtOAc (6:4) to give a light yellow oil (45% yield). ^1^H NMR (CDCl_3_, 293 K): δ 0.91 (t, *J* = 7.6 Hz, 3H); 1.31 (m, 2H); 1.64 (m, 2H); 2.59 (t, *J* = 7.6 Hz, 2H); 7.09 (d, *J* = 6.5 Hz, 2H); 8.46 (d, *J* = 6.5 Hz, 2H).

#### 3.1.14. 4-Butylpiperidine (**22**)

Platinum(IV) oxide (0.2 g, 1 mmol) was added portionwise to a solution of 4-butylpyridine (0.34g, 2.5 mmol) in MeOH (15 mL). A 4 N HCl solution was added to the mixture, and the reaction was stirred in a Parr hydrogenator with 6 atm of H_2_ for 24 h at rt. The catalyst was filtered off, the solvent was evaporated under vacuum, and the residue was diluted with H_2_O and washed with EtOAc (2 × 20 mL). The aqueous layer was basified with 2 N NaOH and extracted with CH_2_Cl_2_ (3 × 20 mL). The organic layer was dried over anhydrous Na_2_SO_4_ and concentrated under reduced pressure to give a colorless oil (72% yield). ^1^H NMR (CDCl_3,_ 293 K): δ 0.89 (t, *J* = 6.7 Hz, 3H); 1.57–1.44 (m, 9H); 1.68 (m, 2H); 2.38 (br s, 1H); 2.59 (m, 2H); 3.09 (m, 2H).

#### 3.1.15. 2-(4-Butylpiperidin-1-yl)ethan-1-ol (**24**)

2-Bromoethanol (0.38 g, 3 mmol) and K_2_CO_3_ (0.6 g, 4 mmol) were added to a solution of **22** (0.3 g, 2.1 mmol) in acetonitrile (10 mL) and the mixture was stirred at 95 °C for 2 h. After cooling down to 25 °C, H_2_O (50 mL) was added, and the solution was extracted with diethyl ether (2 × 30 mL). The organic layer was dried over anhydrous Na_2_SO_4_ and concentrated under reduced pressure to give a residue, which was purified by a flash column eluting with CH_2_Cl_2_/CH_3_OH (9:1) to give an oil (40% yield). This compound was used for the next step without further purification.

#### 3.1.16. 2-(4-Benzylpiperidin-1-yl)ethan-1-ol (**25**)

The procedure described for **24** was used to prepare **25** starting from **23**. A white solid was obtained (45% yield): mp 62–64 °C. It was used for the next step without further purification.

#### 3.1.17. 4-(3-Hydroxypropyl)piperidine (**26**)

The procedure described for **22** was used to prepare **26** starting from 3-(pyridin-4-yl)propan-1-ol. A white solid was obtained (87% yield): mp 58–60 °C. ^1^H NMR (CD_3_OD, 293 K): δ 0.99–1.49 (m, 5H); 1.53–1.63 (m, 2H); 1.74 (m, 2H); 2.59 (m, 2H); 3.04 (m, 2H); 3.56 (t, *J* = 6.6 Hz, 2H).

#### 3.1.18. Ethyl 2-(1-benzylpiperidin-4-ylidene)acetate (**27**)

K_2_CO_3_ (1.38 g, 10 mmol) was added portionwise to a solution of triethyl phosphonoacetate (2.24 g, 10 mmol) in dry THF (20 mL), and the mixture was stirred at reflux for 20 min. After cooling, 1-benzyl-4-piperidone (1.27 g, 6.7 mmol) was added, and the resulting mixture was stirred at reflux for 24 h. After cooling, H_2_O (50 mL) was added, and the mixture was extracted with EtOAc (3 × 30 mL). The organic layer was dried over anhydrous Na_2_SO_4_. The evaporation of the solvent under reduced pressure afforded a residue, which was purified by flash chromatography elution with cyclohexane–EtOAc (9:1) to give a colorless oil (86% yield). ^1^H NMR (CDCl_3_, 293 k): δ 1.27 (t, *J* = 7.2 Hz, 3H); 2.31–2.36 (m, 2H); 2.53 (m, 4H); 2.98 (t, *J* = 5.6 Hz, 2H); 3.53 (s, 2H); 4.15 (q, *J* = 7.2 Hz, 2H); 5.64 (s, 1H); 7.17–7.27 (m, 5H).

#### 3.1.19. 3-(1-Butylpiperidin-4-yl)propan-1-ol (**28**)

A mixture of **26** (0.47 g, 1.7 mmol), 1-bromobutane (0.5 mL), and K_2_CO_3_ (0.29 g, 2 mmol) in acetonitrile (10 mL) was stirred for 3 h at reflux. The evaporation of the solvent under reduced pressure afforded a residue which was diluted with 2 N NaOH and extracted with CH_2_Cl_2_ (3 × 50 mL). The organic layer was dried over anhydrous Na_2_SO_4_. The evaporation of the solvent under reduced pressure afforded a residue, which was purified by flash chromatography elution with 100% EtOAc to give an oil (51% yield). It was used for the next step without further purification.

#### 3.1.20. 3-(1-Benzylpiperidin-4-yl)propan-1-ol (**29**)

The procedure described for **28** was used to prepare **29** starting from **26** and benzyl bromide. An oil was obtained (67% yield). ^1^H NMR (CD_3_OD, 293 K): δ 1.19–1.37 (m, 5H); 1.51–1.62 (m, 2H); 1.72 (m, 2H); 2.02 (m, 2H); 2.92 (m, 2H); 3.52 (s, 2H); 3.55 (t, *J* = 6.7 Hz, 2H); 7.25–7.38 (m, 5H).

#### 3.1.21. 2-(1-Benzylpiperidin-4-yl)ethan-1-ol (**30**)

A solution of **27** (2 g, 7.8 mmol) in dry THF (10 mL) was added dropwise to a suspension of LiAlH_4_ (0.38 g, 10 mmol) in dry THF (10 mL) at 0 °C and the mixture was refluxed for 20 h. After cooling to rt, unreacted LiAlH_4_ was quenched by the careful addition of 10% NaOH solution (8 mL). The mixture was filtered and the residue was diluted with H_2_O, and the solution was extracted with EtOAc (3 × 30 mL). The organic layer was dried over anhydrous Na_2_SO_4_ and concentrated to give a colorless oil (89% yield). ^1^H NMR (CDCl_3_, 293 K): δ 1.34–1.53 (m, 5H); 1.95 (m, 2H); 1.98 (m, 2H); 2.90 (m, 2H); 3.52 (s, 2H); 3.70 (t, *J* = 6.6 Hz, 2H); 7.30–7.36 (m, 5H).

#### 3.1.22. 1-(3-Bromopropyl)-3,4-dihydroquinolin-2(1*H*)-one (**32**)

NaH 60% dispersion in mineral oil (1.36 g, 34 mmol) was added portionwise to a solution of 3,4-dihydroquinolin-2(1*H*)-one (5 g, 34 mmol) in DMF (15 mL). After stirring for 10 min, a solution of 1,3-dibromopropane (7.39 g, 37 mmol) in DMF (10 mL) was added and the resulting mixture was stirred at rt for 20 min. Then, it was diluted with brine (15 mL) and extracted with EtOAc (3 × 20 mL). The organic layer was washed with brine (5 × 20 mL) and dried over anhydrous Na_2_SO_4_. The evaporation of the solvent under reduced pressure afforded a residue, which was purified by flash chromatography with cyclohexane–EtOAc (9:1) to give a colorless oil (73% yield). ^1^H NMR (CDCl_3_, 293 K): δ 2.24 (m, 2H); 2.65 (m, 2H); 2.90 (m, 2H); 3.48 (t, *J* = 6.5 Hz, 2H); 4.09 (m, 2H); 7.30–6.97 (m, 4H).

#### 3.1.23. 1-(4-Bromobutyl)-3,4-dihydroquinolin-2(1*H*)-one (**33**)

The procedure described for **32** was used to prepare **33** starting from 1,4-dibromobutane and 3,4-dihydroquinolin-2(1*H*)-one. An oil was obtained (76% yield). ^1^H NMR (CDCl_3_, 293 K): δ 1.77 (m, 2H); 1.97 (m, 2H); 2.60 (t, *J* = 8 Hz, 2H); 2.85 (t, *J* = 8 Hz, 2H); 3.40 (m, 2H); 3.93 (m, 2H); 7.23–6.96 (m, 4H).

#### 3.1.24. 1-(5-Bromopentyl)-3,4-dihydroquinolin-2(1*H*)-one (**34**)

The procedure described for **32** was used to prepare **34** starting from 1,5-dibromopentane and 3,4-dihydroquinolin-2(1*H*)-one. An oil was obtained (81% yield). ^1^H NMR (CDCl_3_, 293 K): δ 1.52 (m, 2H); 1.68 (m, 2H); 1.94 (m, 2H); 2.63 (t, *J* = 7.8 Hz, 2H); 2.91 (t, *J* = 7.8 Hz, 2H); 3.40 (m, 2H); 3.95 (m, 2H); 6.96–7.23 (m, 4H).

### 3.2. D2-like Radioligand Binding Assays

Radioligand competition binding assays were performed according to a previously reported protocol [40].

### 3.3. BRET Functional Assays

BRET functional assays were performed according to a previously reported protocol [40].

### 3.4. Experimental Details of Modeling Studies

Molecular docking studies were performed considering the resolved D4R structure in complex with nemonapride (PDB id: 5WIU). The protein structure was prepared as previously described in [41]. The ligands were ionized, considering the physiological pH 7.4, and their 3D structures were optimized in VEGA ZZ suite [42] by using semi-empirical PM7-based structural refinement, as implemented in the MOPAC2016 software [43]. Docking studies were carried out by using PLANTS v1.2 [44], selecting a 10Å radius sphere around the co-crystallized ligand as the binding pocket, generating 10 docked poses for each ligand, and setting the ChemPLP scoring function.

The MD simulations involved two putative complexes, as obtained by previous molecular docking experiments for two promising compounds (**12** and **16**), by using Amber v20 software [45]. The Membrane Builder tool by CHARMM-GUI (https://www.charmm-gui.org/ accessed on 11 April 2025) [46] was utilized to generate the protein–membrane system [47]. The protein was parametrized by ff14SB forcefield [48], while Lipid21 [49] was used to parametrize the lipidic components (membrane composed by 70% of POPC and 30% of cholesterol) plus the general forcefield gaff for the ligands [50]. The simulated system was solvated in a box of TIP3P water molecules and neutralized, adding a proper amount of Na^+^ and Cl^-^ ions to achieve a physiological salt concentration of 0.15 M. The rotational and translational positions of the protein in the membrane were calculated by the OPM database (PPM 2.0 server) [51] to model the oriented structure.

After an energy minimization of 10,000 step, a heating phase of 20 ps was carried out, with the temperature increased from 0 K to 300 K by using the Langevin thermostat [52] and by applying positional restraints on the Cα atoms (5 kcal/mol × Å^2^). Then, the system equilibration was performed in the NPT ensemble, maintaining the pressure around 1 bar by using the Berendsen barostat [53]. This phase lasted 100 ps, restraining Cα atoms, whose weight was gradually reduced and removed in the following 140 ps.

Finally, a 200 ns production run was simulated at constant pressure, with the SHAKE algorithm applied [54] and using a timestep of 2 fs. The analyses of the MD trajectories were performed by using the cpptraj module of Amber v22 [55] to calculate RMSD and RMSF profiles.

### 3.5. Experimental Details of Biological Studies in GBM Cell Lines

#### 3.5.1. Cell Lines

Human glioblastoma U87 MG (wild-type TP53 with negative MGMT), T98G (mutant-type TP53 with positive MGMT), and U251 MG (mutant-type TP53 and negative MGMT) cell lines, purchased from Cytion CLS Cell Lines Service (GmbH, Eppelheim, Germany), were maintained at 37 °C with 5% CO_2_ and 95% humidity in Eagle’s minimum essential medium (EMEM) (Euroclone, Milan, Italy) supplemented with heat-inactivated fetal bovine serum (FBS, 10% *v*/*v*), penicillin (100 IU/mL), streptomycin (100 μg/mL), 2 mM L-glutamine, nonessential amino acids (1% *v*/*v*), and sodium pyruvate (1% *v*/*v*).

#### 3.5.2. SRB Assay for Cell Viability

The SRB test was performed to evaluate cell viability. Briefly, 3 × 10^4^ cells/mL were seeded on 96-well plates; the day after, they were treated with 5% CO_2_ and 95% humidity for 48 h at 37 °C, with different concentrations of compounds **12**, **16,** and temozolomide (TMZ, Merk Life Science S.r.l., Milan, Italy) dissolved in DMSO used as the vehicle (V). After treatment, the SRB assay protocol, according to the NCI-60 Screening Methodology provided by the National Cancer Institute, was applied. Finally, the absorbance was quantified at 515 nm with SpectraMax iD3 Multi-Mode Microplate Readers (Molecular Device, San Jose, CA, USA). Cell growth inhibition of 50% (GI_50_) was calculated by [(Ti − Tz)/(C − Tz)] × 100 = 50.

#### 3.5.3. Measuring Cell Death by PI Staining

PI uptake was used to evaluate cell death. Cells (2.5 × 10^4^ cells/mL) seeded on a 12-well plate were treated for 48 h with compounds **12** and **16** or vehicle, at GI_50_ dose. Then, glioma cells were incubated with PI (2 μg/mL) for 30 min at 37 °C. After washing, PI fluorescence was analyzed using a BD Accuri C6 plus flow cytometer and the BD Accuri C6 plus software (BD Biosciences, Milan, Italy). The mean fluorescence intensity (MFI) was assessed. In addition, early apoptosis was evaluated by using an Annexin V-FITC Early Apoptosis Detection Kit followed by citofluorimetric analysis (Cell Signaling Technology, Leiden, The Netherlands) in glioma cells treated for 24 h as described above.

#### 3.5.4. ROS Detection

Intracellular ROS generation was investigated by using a DCFDA (Merk Life Science) probe and flow cytometric analysis. Cells (2.5 × 10^4^ cells/mL) were seeded on a 12-well plate and compounds **15** and **16** or the vehicle were added for 24 and 48 h. Cells were then stained with DCFDA for 20 min at 37 °C, 5% CO_2_, washed, and analyzed by a BD Accuri C6 Plus (BD Biosciences, Milan, Italy). The mean fluorescence intensity (MFI) was assessed.

#### 3.5.5. Mitochondrial Transmembrane Potential (∆Ψm)

Mitochondrial membrane potential in glioma cells was estimated by the staining with JC-1, a cationic carbocyanine dye that accumulates in the mitochondria and is sensitive to mitochondrial membrane potential (Thermo Fisher Scientific, Waltham, MA USA). In normal mitochondria, JC-1 aggregates in the mitochondrial matrix and emits strong red fluorescence (FL-2); when the mitochondrial membrane potential is reduced, JC-1 does not aggregate and produces green fluorescence (FL-1).

Cells (2.5 × 10^4^ cells/mL) were seeded on a 12-well plate and compounds **12** and **16** or the vehicle, at GI_50_ dose, were added for 24 and 48 h. Samples were then analyzed using BD Accuri C6 plus flow cytometer. The MFI of the FL1 and FL2 fluorescence was assessed and the FL2/FL1 ratio was calculated.

#### 3.5.6. Cell Cycle Analysis

Cell cycle analysis involves determining the number of cells in G0/G1, S and G2/M phases by measuring the amount of DNA contained in the nucleus. Cells (2.5 × 10^4^ cells/mL) were plated onto 12-well plates and treated with compounds **12**, **16** or vehicle at GI_50_ dose for 48 h. Then, the cells were harvested and fixed in ice-cold 70% ethanol at 4 °C overnight. The fixed cells were treated with RNase (100 μg/mL) at 37 °C for 30 min and stained with propidium iodide (PI, 20 μg/mL) at 4 °C in the dark for 30 min before measurement.

#### 3.5.7. Colony Formation Assay

After plating glioma cells in 6-well plates (500 cells/well) overnight, compounds **12** and **16** or the vehicle were added for 48 h. At the end of the treatment, the medium was replaced every 3 days for up to two weeks. After that, the cells were fixed with 4% paraformaldehyde for 15 min, washed with PBS, and finally stained with 0.1% crystal violet for 15 min. The stained colonies were counted under the microscope and compared with the vehicle-treated samples. The colony area percentage was calculated by ImageJ software (Version 1.54g).

#### 3.5.8. Statistical Analysis

GraphPad Prism 9.0.1(128) software (GraphPad Software, San Diego, CA, USA) was used for statistical analysis. One-way analysis of variance (ANOVA), Student’ *t* test, and the Shapiro–Wilk test were performed. The results represent the mean ± standard error of the mean (SEM) of three experiments. (* *p* < 0.05, ** *p* < 0.01, *** *p* < 0.001, **** *p* < 0.0001).

## 4. Conclusions

In the present study, new piperidine-based ligands, analogs of the potent and selective D4R compounds 77-LH-28-1 (**7**) and its 4-benzyl analog **8**, were synthesized and studied with the aim of investigating the effects produced by variations in the distances between the pharmacophoric features, such as the quinolinone portion, the basic function, and the butyl substituent or the aromatic terminal, on D4R affinity and selectivity. Radioligand binding results, supported by docking studies and MD simulations, highlighted that the derivatives bearing a terminal butyl chain show SARs different from those with a benzyl terminal, as both shortening of the linker from 3 to 2 carbon chain and shifting of the piperidine nitrogen atom from position 1 to position 4 produce different effects on D4R affinity and selectivity. Specifically, the lower homolog and the regioisomer of the lead **7** (compounds **9** and **15**, respectively) showed a marked decrease in affinity, especially for D4R, while, surprisingly, the same modifications performed in lead **8** (compounds **12** and **16**, respectively) maintained or even increased the high D4R affinity and selectivity. In the functional studies, the response profiles of both compounds **12** and **16** were generally more robust in antagonist mode, with derivative **16** showing higher antagonist potency than **12** across all three transducers, with an inhibition efficacy slightly higher at the Gi protein with respect to the Go protein and β-arrestin 2. Interestingly, **12** and **16** induced a significant dose-dependent decrease in the cell viability of human U87 MG, T98G, and U251 MG glioma cells, as assessed by the SRB assay after 48 h of treatment, with GI_50_ values significantly lower than the first-line chemotherapeutic drug TMZ. Tested at their GI_50_ values, **12** and **16** induced cell death and cell cycle arrest in all three GBM cell lines, promoting an increase in ROS production, encouraging mitochondrial dysfunction, and significantly inhibiting the colony formation. The promising biological profiles of **12** and **16** make them new lead candidates that warrant further investigation to gain a better understanding of the mechanism behind their antitumor activity and to better evaluate their potential for GBM treatment.

## Figures and Tables

**Table 1:** Affinity constants, expressed as p*K*_i_ ^a^ of compounds **7**–**20** for human cloned D2R, D3R, and D4R expressed in HEK293 cells.

Compound	Structure	p*K*_i_			D2R/ D4R	D3R/ D4R
		D2R	D3R	D4R		
**7**	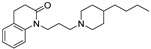	6.17 ± 0.16	6.21 ± 0.13	9.01 ± 0.04	691	631
**8**	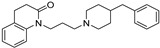	6.04 ± 0.06	5.74 ± 0.28	8.90 ± 0.19	724	1445
**9**	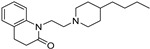	5.05 ± 0.08	4.88 ± 0.05	7.76 ± 0.11	512	758
**10**	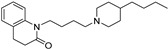	5.87 ± 0.04	6.51 ± 0.10	7.63 ± 0.08	58	13
**11**	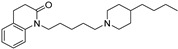	6.52 ± 0.09	6.54 ± 0.07	7.28 ± 0.04	5.8	5.5
**12**	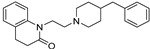	6.18 ± 0.12	6.03 ± 0.04	9.18 ± 0.08	1000	1413
**13**	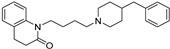	5.97 ± 0.08	6.52 ± 0.03	7.65 ± 0.13	48	13
**14**	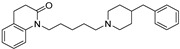	6.09 ± 0.09	6.39 ± 0.12	7.34 ± 0.06	18	8.9
**15**	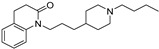	4.99 ± 0.06	5.97 ± 0.07	6.12 ± 0.06	13	1.4
**16**	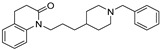	5.44 ± 0.03	5.70 ± 0.12	8.79 ± 0.15	2239	1230
**17**	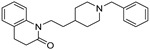	4.90 ± 0.08	5.09 ± 0.05	6.52 ± 0.10	42	27
**18**	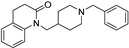	4.88 ± 0.07	5.12 ± 0.10	6.56 ± 0.05	48	28
**19**	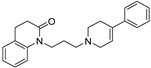	6.24 ± 0.07	6.61 ± 0.11	8.82 ± 0.04	380	162
**20**	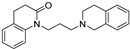	6.35 ± 0.03	6.64 ± 0.08	7.55 ± 0.13	16	8.1

^a ^p*K*_i_ calculated from *K*_i_ values determined by competitive inhibition of [^3^H]*N*-methylspiperone binding in membranes harvested from HEK293 cells stably expressing hD2R, hD3R, or hD4.4R. All values are presented as arithmetic mean ± SEM.

**Table 2:** Potency (expressed as pEC_50_ or pIC_50_) and efficacy values (%Emax or Imax) of dopamine, L745,870, **12** and **16** for D4R expressed in HEK293T cells.

		Dopamine	L745,870	12	16
**pEC_50_** **(pIC_50_ for antagonists)**	**Go**	8.29 ± 0.05	(7.08 ± 0.16)	N.D. (6.67 ± 0.08)	8.36 ± 0.37 (7.21 ± 0.15)
	**Gi**	8.05 ± 0.10	(6.83 ± 0.21)	10.37 ± 1.64 (6.90 ± 0.29)	10.53 ± 2.1 (7.41 ± 0.23)
	**βArr2**	7.57 ± 0.17	(7.97 ± 0.23)	N.D. (6.89 ± 0.31)	7.51 ± 0.64 (8.74 ± 0.45)
**Emax** **(Imax for antagonists)**	**Go**	100.0 ± 1.4	(−74.4 ± 4.1)	N.D. (−68.9 ± 2.4)	23.4 ± 2.5 (−46.7 ± 2.6)
	**Gi**	100.0 ± 2.9	(−76.9 ± 5.5)	15.6 ± 3.8 (−67.9 ± 8.3)	15.5 ± 3.7 (−69.9 ± 5.5)
	**βArr2**	100.0 ± 4.9	(−90.9 ± 6.0)	N.D. (−46.6 ± 6.0)	17.9 ± 3.9 (−43.1 ± 5.2)

N.D. = cannot be determined.

**Table 3:** GI_50_^a^ values of compounds **12**, **16** and TMZ measured in U87 MG, T98G and U251 MG human glioma cell lines.

	U87 MG (GI_50_, µM)	T98G (GI_50_, µM)	U251 MG (GI_50_, µM)
**12**	52.9 ± 0.6	25.7 ± 1.9	87.5 ± 1.0
**16**	65.5 ± 1.3	23.0 ± 4.8	90.4 ± 3.3
**TMZ**	>200	>200	>200

^a^ GI_50_: drug concentration (µM) required to inhibit 50% net of cell growth.

## Data Availability

Data are contained within the article.

## Supplementary Materials

The following supporting information can be downloaded at https://www.mdpi.com/article/10.3390/ph18050739/s1, Table S1: Elemental analysis results for compounds **9**–**20**; Figure S1: The effects of compounds **12**, **16**, and TMZ on U87 MG, T98G, and U251 MG glioma cells; Figure S2: Annexin V-FITC and PI staining of glioma cells treated with vehicle or with compounds **12** and **16** for 24 h; Equipment for compounds preparation and characterization.

## Abbreviations

The following abbreviations are used in this manuscript:

DRs	Dopamine receptors
CNS	Central nervous system
ICL3	Third intracellular loop
GBM	Glioblastoma
BRET	Bioluminescence resonance energy transfer
SRB MD	Sulforhodamine B Molecular dynamics
TMZ	Temozolomide
PI	Propidium iodide
ROS	Reactive oxygen species
MFI	Mean fluorescence intensity
DCFDA	2′,7′-Dichlorodihydrofluorescein diacetate
FBS	Fetal bovine serum
PBS	Phosphate-buffer saline

## References

[1] Beaulieu J.M., Gainetdinov R.R. (2011). The physiology, signaling, and pharmacology of dopamine receptors. Pharmacol. Rev..

[2] Xin J., Fan T., Guo P., Wang J. (2019). Identification of functional divergence sites in dopamine receptors of vertebrates. Comput. Biol. Chem..

[3] Beaulieu J.M., Espinoza S., Gainetdinov R.R. (2015). Dopamine receptors—IUPHAR Review 13. Br. J. Pharmacol..

[4] Vallone D., Picetti R., Borrelli E. (2000). Structure and function of dopamine receptors. Neurosci. Biobehav. Rev..

[5] Huff R.M., Chio C.L., Lajiness M.E., Goodman L.V. (1998). Signal transduction pathways modulated by D2-like dopamine receptors. Adv. Pharmacol..

[6] Giorgioni G., Del Bello F., Pavletić P., Quaglia W., Botticelli L., Cifani C., Micioni Di Bonaventura E., Micioni Di Bonaventura M.V., Piergentili A. (2021). Recent findings leading to the discovery of selective dopamine D(4) receptor ligands for the treatment of widespread diseases. Eur. J. Med. Chem..

[7] Botticelli L., Micioni Di Bonaventura E., Del Bello F., Giorgioni G., Piergentili A., Romano A., Quaglia W., Cifani C., Micioni Di Bonaventura M.V. (2020). Underlying Susceptibility to Eating Disorders and Drug Abuse: Genetic and Pharmacological Aspects of Dopamine D4 Receptors. Nutrients.

[8] Boateng C.A., Nilson A.N., Placide R., Pham M.L., Jakobs F.M., Boldizsar N., McIntosh S., Stallings L.S., Korankyi I.V., Kelshikar S. (2023). Pharmacology and Therapeutic Potential of Benzothiazole Analogues for Cocaine Use Disorder. J. Med. Chem..

[9] Lindsley C.W., Hopkins C.R. (2017). Return of D(4) Dopamine Receptor Antagonists in Drug Discovery. J. Med. Chem..

[10] Asghari V., Sanyal S., Buchwaldt S., Paterson A., Jovanovic V., Van Tol H.H.M. (1995). Modulation of Intracellular Cyclic AMP Levels by Different Human Dopamine D4 Receptor Variants. J. Neurochem..

[11] Ariano M.A., Wang J., Noblett K.L., Larson E.R., Sibley D.R. (1997). Cellular distribution of the rat D4 dopamine receptor protein in the CNS using anti-receptor antisera. Brain Res..

[12] Dolma S., Selvadurai H.J., Lan X., Lee L., Kushida M., Voisin V., Whetstone H., So M., Aviv T., Park N. (2016). Inhibition of Dopamine Receptor D4 Impedes Autophagic Flux, Proliferation, and Survival of Glioblastoma Stem Cells. Cancer Cell.

[13] Pavletić P., Semeano A., Yano H., Bonifazi A., Giorgioni G., Piergentili A., Quaglia W., Sabbieti M.G., Agas D., Santoni G. (2022). Highly Potent and Selective Dopamine D4 Receptor Antagonists Potentially Useful for the Treatment of Glioblastoma. J. Med. Chem..

[14] Ye N., Neumeyer J.L., Baldessarini R.J., Zhen X., Zhang A. (2013). Update 1 of: Recent progress in development of dopamine receptor subtype-selective agents: Potential therapeutics for neurological and psychiatric disorders. Chem. Rev..

[15] Wang S., Wacker D., Levit A., Che T., Betz R.M., McCorvy J.D., Venkatakrishnan A.J., Huang X.P., Dror R.O., Shoichet B.K. (2017). D(4) dopamine receptor high-resolution structures enable the discovery of selective agonists. Science.

[16] Zhou Y., Cao C., He L., Wang X., Zhang X.C. (2019). Crystal structure of dopamine receptor D4 bound to the subtype selective ligand, L745870. eLife.

[17] Lyu J., Wang S., Balius T.E., Singh I., Levit A., Moroz Y.S., O’Meara M.J., Che T., Algaa E., Tolmachova K. (2019). Ultra-large library docking for discovering new chemotypes. Nature.

[18] Graßl F., Bock L., Huete-Huerta González Á., Schiller M., Gmeiner P., König J., Fromm M.F., Hübner H., Heinrich M.R. (2023). Exploring Structural Determinants of Bias among D4 Subtype-Selective Dopamine Receptor Agonists. J. Med. Chem..

[19] Levoin N., Murthy A.V.R., Narendar V., Kumar N.S., Aparna P., Bhavani A.K.D., Reddy C.R., Mosset P., Grée R. (2022). Discovery of potent dual ligands for dopamine D4 and σ1 receptors. Bioorganic Med. Chem..

[20] Tolentino K.T., Mashinson V., Vadukoot A.K., Hopkins C.R. (2022). Discovery and characterization of benzyloxy piperidine based dopamine 4 receptor antagonists. Bioorganic Med. Chem. Lett..

[21] Del Bello F., Bonifazi A., Giorgioni G., Cifani C., Micioni Di Bonaventura M.V., Petrelli R., Piergentili A., Fontana S., Mammoli V., Yano H. (2018). 1-[3-(4-Butylpiperidin-1-yl) propyl]-1, 2, 3, 4-tetrahydroquinolin-2-one (77-LH-28-1) as a model for the rational design of a novel class of brain penetrant ligands with high affinity and selectivity for dopamine D4 receptor. J. Med. Chem..

[22] Meyers A.I., Edwards P.D., Rieker W.F., Bailey T.R. (1984). .alpha.-Amino carbanions via formamidines. Alkylation of pyrrolidines, piperidines, and related heterocycles. J. Am. Chem. Soc..

[23] Vila N., Besada P., Viña D., Sturlese M., Moro S., Terán C. (2016). Synthesis, biological evaluation and molecular modeling studies of phthalazin-1(2H)-one derivatives as novel cholinesterase inhibitors. RSC Adv..

[24] Egbertson M.S., Chang C.T.C., Duggan M.E., Gould R.J., Halczenko W., Hartman G.D., Laswell W.L., Lynch J.J., Lynch R.J. (1994). Non-Peptide Fibrinogen Receptor Antagonists. 2. Optimization of a Tyrosine Template as a Mimic for Arg-Gly-Asp. J. Med. Chem..

[25] Contreras J.-M., Parrot I., Sippl W., Rival Y.M., Wermuth C.G. (2001). Design, Synthesis, and Structure–Activity Relationships of a Series of 3-[2-(1-Benzylpiperidin-4-yl)ethylamino]pyridazine Derivatives as Acetylcholinesterase Inhibitors. J. Med. Chem..

[26] Bonifazi A., Newman A.H., Keck T.M., Gervasoni S., Vistoli G., Del Bello F., Giorgioni G., Pavletić P., Quaglia W., Piergentili A. (2021). Scaffold Hybridization Strategy Leads to the Discovery of Dopamine D(3) Receptor-Selective or Multitarget Bitopic Ligands Potentially Useful for Central Nervous System Disorders. ACS Chem. Neurosci..

[27] Del Bello F., Ambrosini D., Bonifazi A., Newman A.H., Keck T.M., Giannella M., Giorgioni G., Piergentili A., Cappellacci L., Cilia A. (2019). Multitarget 1,4-Dioxane Compounds Combining Favorable D(2)-like and 5-HT(1A) Receptor Interactions with Potential for the Treatment of Parkinson’s Disease or Schizophrenia. ACS Chem. Neurosci..

[28] Sazonova E.V., Chesnokov M.S., Zhivotovsky B., Kopeina G.S. (2022). Drug toxicity assessment: Cell proliferation versus cell death. Cell Death Discov..

[29] Nicholson J.G., Fine H.A. (2021). Diffuse Glioma Heterogeneity and Its Therapeutic Implications. Cancer Discov..

[30] Zhao H., Wang C., Lu B., Zhou Z., Jin Y., Wang Z., Zheng L., Liu K., Luo T., Zhu D. (2016). Pristimerin triggers AIF-dependent programmed necrosis in glioma cells via activation of JNK. Cancer Lett..

[31] Zhou J., Li G., Han G., Feng S., Liu Y., Chen J., Liu C., Zhao L., Jin F. (2020). Emodin induced necroptosis in the glioma cell line U251 via the TNF-α/RIP1/RIP3 pathway. Investig. New Drugs.

[32] Pagano C., Navarra G., Coppola L., Avilia G., Pastorino O., Della Monica R., Buonaiuto M., Torelli G., Caiazzo P., Bifulco M. (2022). N6-isopentenyladenosine induces cell death through necroptosis in human glioblastoma cells. Cell Death Discov..

[33] Brunetti A., Marinelli O., Morelli M.B., Iannarelli R., Amantini C., Russotti D., Santoni G., Maggi F., Nabissi M. (2019). Isofuranodiene synergizes with temozolomide in inducing glioma cells death. Phytomedicine.

[34] Liang Z., Zhao S., Liu Y., Cheng C. (2025). The promise of mitochondria in the treatment of glioblastoma: A brief review. Discov. Oncol..

[35] Qin X., Xu Y., Peng S., Qian S., Zhang X., Shen S., Yang J., Ye J. (2020). Sodium butyrate opens mitochondrial permeability transition pore (MPTP) to induce a proton leak in induction of cell apoptosis. Biochem. Biophys. Res. Commun..

[36] Begum H.M., Shen K. (2023). Intracellular and microenvironmental regulation of mitochondrial membrane potential in cancer cells. WIREs Mech. Dis..

[37] Ramesh G., Das S., Bola Sadashiva S.R. (2020). Berberine, a natural alkaloid sensitizes human hepatocarcinoma to ionizing radiation by blocking autophagy and cell cycle arrest resulting in senescence. J. Pharm. Pharmacol..

[38] Huang Y.P., Huang W.W., Tsai K.F., Shiao L.R., Yang Z.H., Tseng S.Y., Lin Y.H., Chen C.Y., Chan P., Leung Y.M. (2023). CDN1163, a SERCA activator, causes intracellular Ca^(2+)^ leak, mitochondrial hyperpolarization and cell cycle arrest in mouse neuronal N2A cells. Neurotoxicology.

[39] Mani S., Swargiary G., Singh K.K. (2020). Natural Agents Targeting Mitochondria in Cancer. Int. J. Mol. Sci..

[40] Matteucci F., Pavletić P., Bonifazi A., Del Bello F., Giorgioni G., Piergentili A., Amantini C., Zeppa L., Sabato E., Vistoli G. (2025). New Arylpiperazines as Potent and Selective Dopamine D4 Receptor Ligands Potentially Useful to Treat Glioblastoma. J. Med. Chem..

[41] Del Bello F., Bonifazi A., Giorgioni G., Piergentili A., Sabbieti M.G., Agas D., Dell’Aera M., Matucci R., Górecki M., Pescitelli G. (2020). Novel Potent Muscarinic Receptor Antagonists: Investigation on the Nature of Lipophilic Substituents in the 5- and/or 6-Positions of the 1,4-Dioxane Nucleus. J. Med. Chem..

[42] Pedretti A., Mazzolari A., Gervasoni S., Fumagalli L., Vistoli G. (2021). The VEGA suite of programs: An versatile platform for cheminformatics and drug design projects. Bioinformatics.

[43] Stewart J.J. (2013). Optimization of parameters for semiempirical methods VI: More modifications to the NDDO approximations and re-optimization of parameters. J. Mol. Model..

[44] Korb O., Stützle T., Exner T.E. (2009). Empirical scoring functions for advanced protein-ligand docking with PLANTS. J. Chem. Inf. Model..

[45] Case D.A., Cheatham T.E., Darden T., Gohlke H., Luo R., Merz K.M., Onufriev A., Simmerling C., Wang B., Woods R.J. (2005). The Amber biomolecular simulation programs. J. Comput. Chem..

[46] Jo S., Kim T., Iyer V.G., Im W. (2008). CHARMM-GUI: A web-based graphical user interface for CHARMM. J. Comput. Chem..

[47] Wu E.L., Cheng X., Jo S., Rui H., Song K.C., Dávila-Contreras E.M., Qi Y., Lee J., Monje-Galvan V., Venable R.M. (2014). CHARMM-GUI Membrane Builder toward realistic biological membrane simulations. J. Comput. Chem..

[48] Maier J.A., Martinez C., Kasavajhala K., Wickstrom L., Hauser K.E., Simmerling C. (2015). ff14SB: Improving the Accuracy of Protein Side Chain and Backbone Parameters from ff99SB. J. Chem. Theory Comput..

[49] Dickson C.J., Walker R.C., Gould I.R. (2022). Lipid21: Complex Lipid Membrane Simulations with AMBER. J. Chem. Theory Comput..

[50] He X., Man V.H., Yang W., Lee T.S., Wang J. (2020). A fast and high-quality charge model for the next generation general AMBER force field. J. Chem. Phys..

[51] Lomize M.A., Pogozheva I.D., Joo H., Mosberg H.I., Lomize A.L. (2012). OPM database and PPM web server: Resources for positioning of proteins in membranes. Nucleic Acids Res..

[52] Paterlini M.G., Ferguson D.M. (1998). Constant temperature simulations using the Langevin equation with velocity Verlet integration. Chem. Phys..

[53] Berendsen H.J.C., Postma J.P.M., van Gunsteren W.F., DiNola A., Haak J.R. (1984). Molecular dynamics with coupling to an external bath. J. Chem. Phys..

[54] Kräutler V., van Gunsteren W.F., Hünenberger P.H. (2001). A fast SHAKE algorithm to solve distance constraint equations for small molecules in molecular dynamics simulations. J. Comput. Chem..

[55] Roe D.R., Cheatham T.E. (2013). PTRAJ and CPPTRAJ: Software for Processing and Analysis of Molecular Dynamics Trajectory Data. J. Chem. Theory Comput..

