# Supplementary material for: Novel Potent and Selective Dopamine D4 Receptor Piperidine Antagonists as Potential Alternatives for the Treatment of Glioblastoma
Source: Pharmaceuticals (Basel). 2025 May 17;18(5):739. doi: 10.3390/ph18050739 (PMC12114630; doi:10.3390/ph18050739)
Supplement: Supplementary file 1 [file pharmaceuticals-18-00739-s001.zip › pharmaceuticals-3563745-supplementary.pdf]

SUPPLEMENTARY MATERIALS

Novel Potent and Selective Dopamine D4 Receptor Piperidine Antagonists as Potential Alternatives for the Treatment of Glioblastoma

Federica Matteucci[1,†], Pegi Pavletić[1,2,†], Alessandro Bonifazi[3], Rian Garland[4], Hideaki Yano[4], Consuelo Amantini[5], Laura Zeppa[5], Emanuela Sabato[6], Giulio Vistoli[6], Valerio Mammoli[7], Loredana Cappellacci[1], Fabio Del Bello[1,*], Gianfabio Giorgioni[1], Riccardo Petrelli[1], Alessia Piergentili[1], Wilma Quaglia[1,*] and Alessandro Piergentili[1]

[1] School of Pharmacy, Medicinal Chemistry Unit, Chemistry Interdisciplinary Project (ChIP), University of Camerino, via Madonna delle Carceri, 62032 Camerino, Italy
[2] Faculty of Biotechnology and Drug Development, University of Rijeka, Radmile Matejcic 2, 51000 Rijeka, Croatia
[3] Department of Pharmacology and Toxicology, Center for Addiction Sciences and Therapeutics, University of Texas Medical Branch, Galveston, TX 77555, USA
[4] Department of Pharmaceutical Sciences, School of Pharmacy and Pharmaceutical Sciences, Bouvé College of Health Sciences, Center for Drug Discovery, Northeastern University, Boston, MA 02115, USA
[5] School of Biosciences and Veterinary Medicine, Immunopathology and Molecular Medicine Unit, University of Camerino, via Madonna delle Carceri 9, 62032 Camerino, Italy
[6] Department of Pharmaceutical Sciences, University of Milan, Via Mangiagalli 25, 20133 Milano, Italy
[7] Center for Drug Discovery and Development-IDD, Aptuit, an Evotec company, Via A. Fleming, 4, 37135 Verona, Italy

[*] Correspondence: fabio.delbello@unicam.it, Tel.: +39- 0737-402265; wilma.quaglia@unicam.it, Tel.: +39-0737-402237
[†] These authors contributed equally to this work.

Table of Contents:

Table S1: Elemental analysis results for compounds **9-20**..………..…………………...….……S2

Figure S1: The effects of compounds **12**, **16**, and TMZ on cell viability of U87 MG, T98G and U251 MG glioma cells as evaluated by SRB assay…………………………………………...…S3

Figure S2: Annexin V-FITC and PI staining of glioma cells treated with vehicle or with compounds **12** and **16** for 24 h…………...………..………………………………………………..S4

Equipment for compounds preparation and characterization…………………………………S5

Table S1. Elemental analysis results for compounds **9–20**.

**Compd**	**Formula**	**Calcd**			**Found**		
		**C%**	**H%**	**N%**	**C%**	**H%**	**N%**
**9**	$C_{20}H_{30}N_2O.C_2H_2O_4$	65.32	7.97	6.93	65.70	8.11	6.80
**10**	$C_{22}H_{34}N_2O.C_2H_2O_4$	66.64	8.39	6.48	66.45	8.48	6.22
**11**	$C_{23}H_{36}N_2O.C_2H_2O_4$	67.24	8.58	6.27	66.99	8.34	6.50
**12**	$C_{23}H_{28}N_2O.C_2H_2O_4$	68.47	6.90	6.39	68.77	6.97	6.11
**13**	$C_{25}H_{32}N_2O.C_2H_2O_4$	69.51	7.35	6.00	69.19	7.67	5.87
**14**	$C_{26}H_{34}N_2O.C_2H_2O_4$	69.98	7.55	5.83	69.75	7.65	5.61
**15**	$C_{21}H_{32}N_2O.C_2H_2O_4$	66.00	8.19	6.69	66.32	8.00	6.90
**16**	$C_{24}H_{30}N_2O.C_2H_2O_4$	69.01	7.13	6.19	68.72	7.33	6.02
**17**	$C_{23}H_{28}N_2O.C_2H_2O_4$	68.47	6.90	6.39	68.20	7.03	6.48
**18**	$C_{22}H_{26}N_2O.C_2H_2O_4$	67.91	6.65	6.60	67.69	6.88	6.42
**19**	$C_{23}H_{26}N_2O.C_2H_2O_4$	68.79	6.47	6.42	68.38	6.39	6.67
**20**	$C_{21}H_{24}N_2O.C_2H_2O_4$	67.30	6.38	6.82	67.07	6.19	6.98

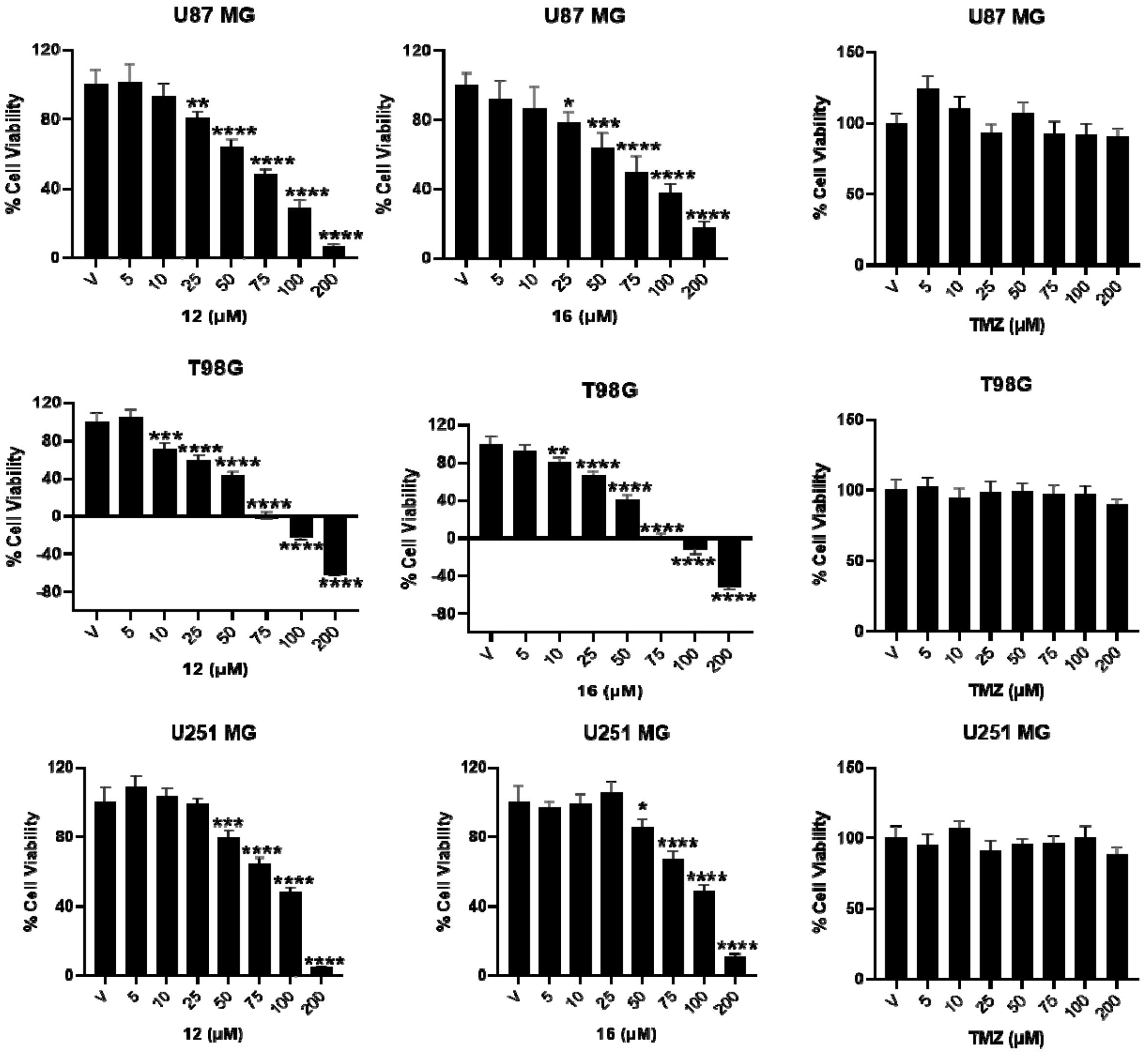

Figure S1. SRB assay was used to evaluate cell viability in glioma cells treated with compounds **12**, **16**, or vehicle for 48 h. TMZ was used as reference since it is the main drug used in cerebral cancers. Data represent the mean ± SEM of three separate experiments. * $p < 0.05$; ** $p < 0.01$; *** $p < 0.001$; **** $p < 0.0001$.

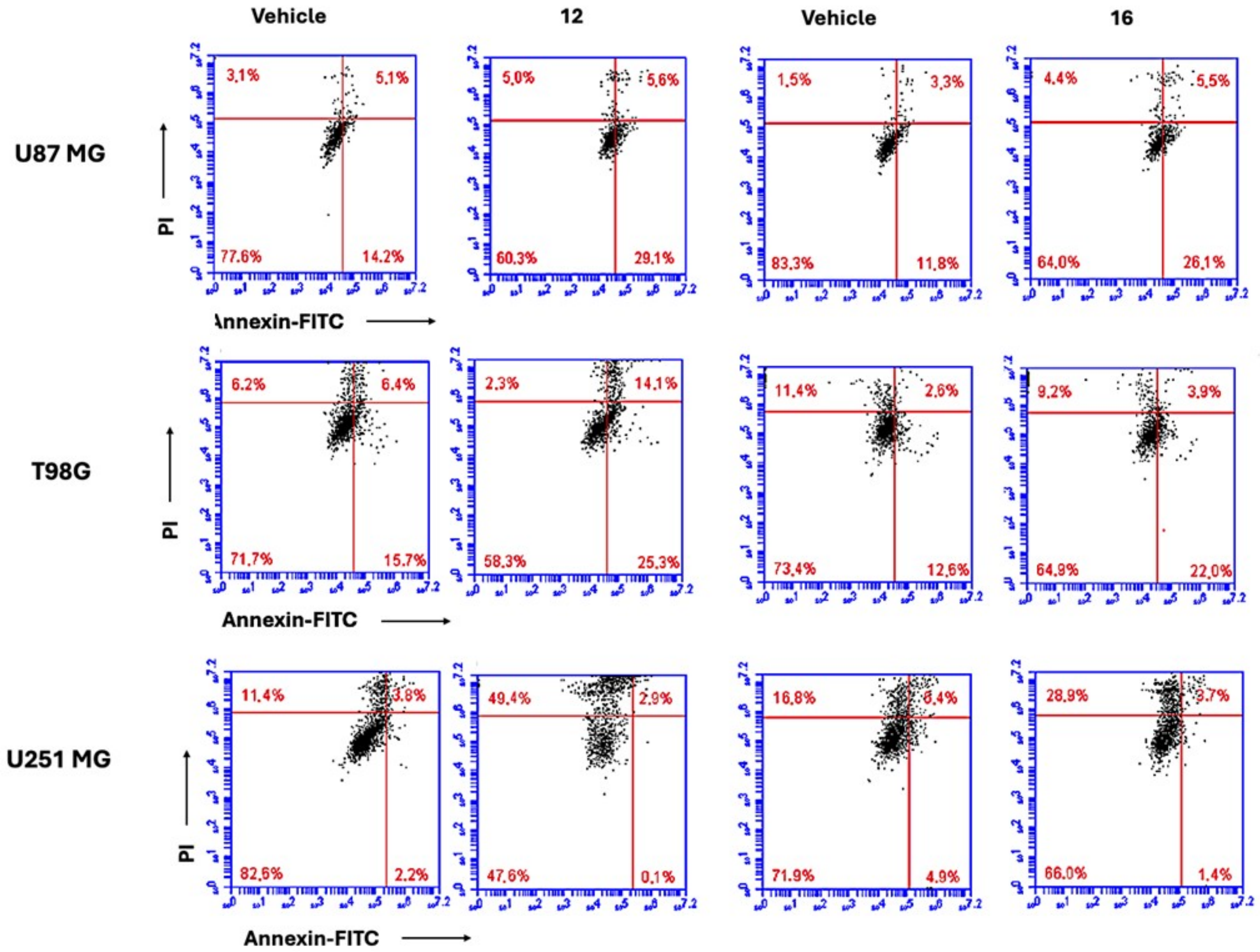

Figure S2. Annexin V-FITC and PI staining of glioma cells treated with vehicle or with compounds **12** and **16** for 24 h. Data are representative of three separate experiments. Numbers in each single quadrant represent the percentage of cells.

Equipment for compounds preparation and characterization.

Melting points were taken in glass capillary tubes on a Büchi SMP-20 apparatus (Büchi Laboratory Equipment, Uster, Switzerland) and are uncorrected. ^{1}H-NMR spectra were recorded either with a Bruker 500 Ascend (Bruker BioSpin Corporation, Billerica, MA USA) and Varian Mercury AS400 instruments (Varian Srl, Palo Alto, CA USA), and chemical shifts (ppm) are reported relative to tetramethylsilane. Spin multiplicities are given as s (singlet), d (doublet), dd (double doublet), t (triplet), or m (multiplet). IR spectra were recorded on PerkinElmer 297 instrument (PerkinElmer Inc., Waltham, MA USA) and spectral data (not shown because of the lack of unusual features) were obtained for all compounds reported and are consistent with the assigned structures. The microanalyses were recorded on FLASH 2000 instrument (ThermoFisher Scientific, Waltham, MA USA). The elemental composition of the compounds agreed to within ±0.4% of the calculated value. All reactions were monitored by thin-layer chromatography using silica gel plates (60 F254; Merck), visualizing with ultraviolet light. Chromatographic separations were performed on silica gel columns (Kieselgel 40, 0.040−0.063 mm, Merck) by flash chromatography. Compounds were named following IUPAC rules as applied by ChemBioDraw Ultra (version 12.0) software for systematically naming organic chemicals. The purity of the novel compounds was determined by combustion analysis and was ≥95%.
